# Task context load induces reactive cognitive control: An fMRI study on cortical and brain stem activity

**DOI:** 10.3758/s13415-019-00691-6

**Published:** 2019-01-18

**Authors:** Veronica Mäki-Marttunen, Thomas Hagen, Thomas Espeseth

**Affiliations:** 1 Department of Psychology, University of Oslo, Postbox 1094, 0317 Oslo, Norway; 2 Norwegian Centre for Mental Disorders Research (NORMENT), KG Jebsen Centre for Psychosis Research, Division of Mental Health and Addiction, Ullevål Hospital, Building 49, Kirkeveien 166, Postbox 4956, 0424 Oslo, Norway

**Keywords:** Attention, Cognitive control, AX–continuous performance task, Locus coeruleus, Ventral tegmental area/substantia nigra, fMRI

## Abstract

Cognitive control is a highly dynamic process that relies on flexible engagement of prefrontal areas and of neuromodulatory systems in order to adapt to changing demands. A range of internal and external factors come into play when individuals engage in a task requiring cognitive control. Here we investigated whether increased working memory (WM) demands would induce a flexible change in cognitive control mode in young healthy individuals. We developed a novel variant of the well-known AX–continuous performance task (AX-CPT). We manipulated the cognitive demands of maintaining task-relevant contextual information and studied the impact of this manipulation on behavior and brain activity. We expected that low WM load would allow for a more effortful, proactive strategy, while high WM load would induce a strategy of less effortful, stimulus-driven reactive control. In line with our hypothesis, a web-based experiment revealed that increased load was associated with more reactive behavioral responses, and this finding was independently replicated in behavioral data acquired in the MRI scanner. The results from brain activity showed that the right dorsolateral prefrontal cortex was activated by cues in the proactive mode and by probes in the reactive mode. The analysis of task-induced brain stem activity indicated that both the dopaminergic and noradrenergic systems are involved in updating context representations, and that, respectively, these systems mediate a gating signal to the control network and are involved in the dynamic regulation of task engagement.

**Electronic supplementary material:**

The online version of this article (10.3758/s13415-019-00691-6) contains supplementary material, which is available to authorized users.

Humans have an unrivalled ability to define a future goal and to plan and carry out a sequence of thoughts or actions to reach it. This ability is specifically linked to cognitive control—that is, the capacity to use contextual representations (e.g., instructions, evaluation of utility, etc.) to guide thoughts and actions in accordance with goals (Badre & Nee, 2018; Braver, 2012; Duncan, Emslie, Williams, Johnson, & Freer, 1996; Engle & Kane, 2004; Miller & Cohen, 2001; Munakata, Morton, & O’Reilly, 2007; Smith & Jonides, 1999). Cognitive control can often be implemented by using different strategies, which seem to vary from situation to situation and from individual to individual. Indeed, the existence of different cognitive strategies has been argued to be an intrinsic and naturally occurring property of higher cognitive functions (Evans & Stanovich, 2013; Jacoby, Kelley, & McElree, 1999; Stanovich & West, 2000). One remarkable feature of cognitive control is its *adaptive flexibility*—that is, its capability to update goals and strategies when internal or environmental factors indicate that it may be advantageous to do so. Adaptive flexibility allows the brain to update the context according to the task at hand and to internal physiological states. Consistent with this view, the dual mechanisms of control (DMC) framework suggests that cognitive control may operate in either proactive or reactive control modes (Braver, 2012; Braver, Gray, & Burgess, 2007). Whereas *proactive* control refers to anticipatory and sustained maintenance of goal representations (i.e., the context), *reactive* control reflects transient stimulus-driven reactivation of goal representations.

A central assumption in the DMC framework is that variability in cognitive control is determined by the dynamic balance between proactive and reactive processes. Importantly, many factors, including variables that vary over time or from person to person, can potentially contribute to the weighting of proactive versus reactive processes in a particular task. Thus, individual differences in such characteristics as age, cognitive abilities, and the presence of neuropsychiatric disorders influence cognitive control strategy, but so do subtle changes in situational factors, such as changes in task demands and reward contingencies, or transcranial direct current stimulation (tDCS) during task performance (Gómez-Ariza, Martín, & Morales, 2017). One factor that influences the implementation of cognitive control is the availability of resources in a participant’s working memory (WM) to do the task while also analyzing cues so as to prepare for upcoming challenges. For example, Speer, Jacoby, and Braver (2003) manipulated the expectation of subsequent WM load and observed a more reactive strategy associated with high load expectation. Marklund and Persson (2012) used a three-back task, which requires participants to employ a reactive strategy. The task is highly demanding, and presenting cues that function to warn participants of forthcoming high-interference or high-retention-load tasks induced a more proactive strategy in the participants and enabled them to better maintain performance level. Working memory is generally assumed to have limited capacity (Anderson, 1983; Cohen, Dunbar, & McClelland, 1990; Cowan, 2005; Luck & Vogel, 1997; G. A. Miller, 1956), and the finding that high stimulus interference or high retention load is associated with a reactive processing mode shows that the proactive mode requires more WM resources than the reactive mode. Consistent with this, children, old adults, and individuals with neuropsychiatric disorders (groups that are known to have limited WM capacities), tend to employ reactive modes of cognitive control. However, a more proactive processing mode could be induced by reward or strategic training in these groups (Braver, 2012; Braver, Paxton, Locke, & Barch, 2009). Thus, in summary, the literature shows that tasks that require WM resources are associated with reactive processing, but that a more proactive processing mode can be induced by informative cues, rewards, or strategic training. In contrast, we aimed to induce a more reactive processing mode by increasing context load in a task where young healthy participants typically employ a proactive processing strategy.

The AX-CPT task (a version of the classic continuous performance test; Rosvold, Mirsky, Sarason, Bransome, & Beck, 1956) is one of the cognitive control tasks most frequently used to study context updating by cognitive and clinical neuroscientists (Cohen & Servan-Schreiber, 1992; Servan-Schreiber, Cohen, & Steingard, 1996). In this task, young healthy adults typically present a proactive mode of control, while various developmental and neuropsychiatric groups tend to employ a reactive control mode (Chatham, Frank, & Munakata, 2009; Lesh et al., 2013). Low WM capacity (WMC) also predicts a predominance of a reactive processing mode in healthy young individuals that are within the normal range of WMC scores (Redick, 2014; Richmond, Redick, & Braver, 2015; Wiemers & Redick, 2018). In the present study, we aimed to develop a version of AX-CPT that can induce a reactive processing mode in healthy young participants. We did so by increasing load in the task context. Furthermore, we aimed to study the temporal dynamics of cortical and brain stem activity associated with task performance. Our major questions were: Can task context load induce a reactive control mode? And if it does so, can this manipulation mimic the behavioral and brain activity effects observed in children, old adults, low-WMC controls and various patient groups?

At the neural level, the representation of context is strongly linked to the dorsolateral prefrontal cortex (dlPFC) and associated systems (Badre & Nee, 2018; Cohen & Servan-Schreiber, 1992; Miller & Cohen, 2001; Nee, Jahn, & Brown, 2013). The use of context in the AX-CPT during proactive control relies on sustained cue-related brain activity in the trials where the cues predict the response regardless of the probe (B cues). Dorsolateral prefrontal cortex and midbrain nuclei have been implicated in this sustained activity (Barch et al., 1997; Braver & Barch, 2002; Braver & Bongiolatti, 2002; Braver et al., 2009; D’Ardenne et al., 2012). On the other hand, during reactive behavior, individuals do not actively maintain contextual information and the dorsolateral prefrontal cortex shows reduced sustained activity (Edwards, Barch, & Braver, 2010; Lesh et al., 2013; Paxton, Barch, Racine, & Braver, 2007). It has been suggested that an impairment in the activity of this region, particularly on the right side, may relate to cognitive deficits in schizophrenia (Barch & Ceaser, 2012; Davidson & Heinrichs, 2003; Glahn et al., 2005; Goldman-Rakic & Selemon, 1997; Van Snellenberg, Torres, & Thornton, 2006).

With regard to how the prefrontal cortex updates context, one hypothesis is that it uses a dopaminergic gating mechanism—that is, a propagation of a contextual update signal to frontal cognitive control circuits (Badre & Nee, 2018; Braver & Cohen, 2000; Frank, Loughry, & O’Reilly, 2001; Rougier, Noelle, Braver, Cohen, & O’Reilly, 2005). In the absence of such a signal, inputs have weak influence on dlPFC, allowing representations that are currently active to persist. When the gating signal occurs, inputs to the dlPFC are enhanced. For example, D’Ardenne et al. (2012) showed that dlPFC is causally involved in representing the currently relevant context, and that the dopaminergic system is involved in gating an update signal to the dlPFC. In particular, phasic activation of the ventral tegmental area/substantia nigra (VTA/SN) was temporally associated with dlPFC activity, but only in conditions in which context updating was required.

Another central variable in the adaptive flexibility of control is cognitive effort, or the intensity of task engagement. The degree of task engagement is measured by improved speed and/or accuracy driven by norepinephrine (NE)-induced modulation of neural gain (Aston-Jones & Cohen, 2005). Neural gain is implicated in cognitive control functions such as WM, task switching, and response inhibition via neural dynamics in the PFC (Robbins & Arnsten, 2009). There is increasing evidence that effort can be measured as increased activity in the locus coeruleus (LC; Alnaes et al., 2014). However, few studies have directly examined the involvement of the LC-NE system in the flexible nature of cognitive control (Köhler, Bär, & Wagner, 2016). The study of LC activity in humans presents technical challenges due to its small size, the difficulty in locating its position (which may vary significantly across individuals) and its susceptibility to contamination by artifacts. To overcome these challenges, we obtained structural magnetic resonance imaging (MRI) sequences specifically designed to identify neuromelanin-rich regions in the brain, as is the case of the LC (Keren, Lozar, Harris, Morgan, & Eckert, 2009). This constituted a technical advantage that allowed us to obtain reliable signals from the LC of each participant.

To summarize, we pursued several goals: to (1) study whether a load manipulation in the task context of the AX-CPT induces reactive behavior in healthy adults, (2) study whether load demands influence the dynamics of brain activity in cognitive control areas, (3) investigate whether the VTA/SN and LC are involved in context processing and load manipulations, and (4) compare individuals with different proactivity patterns in terms of behavioral, cortical and brain stem activity profiles under low and high load. We hypothesized that task context load would induce a shift from proactive to reactive behavior and from sustained to transient activity in areas associated with context processing, in line with the DMC framework (Braver et al., 2009; Paxton et al., 2007; see Fig. 1). In particular, given the literature reviewed above, we expected this effect to be more pronounced in the prefrontal cortex and VTA/SN. We expected to find a similar pattern in LC, consistent with its role in the allocation of effort.

**Fig. 1:**
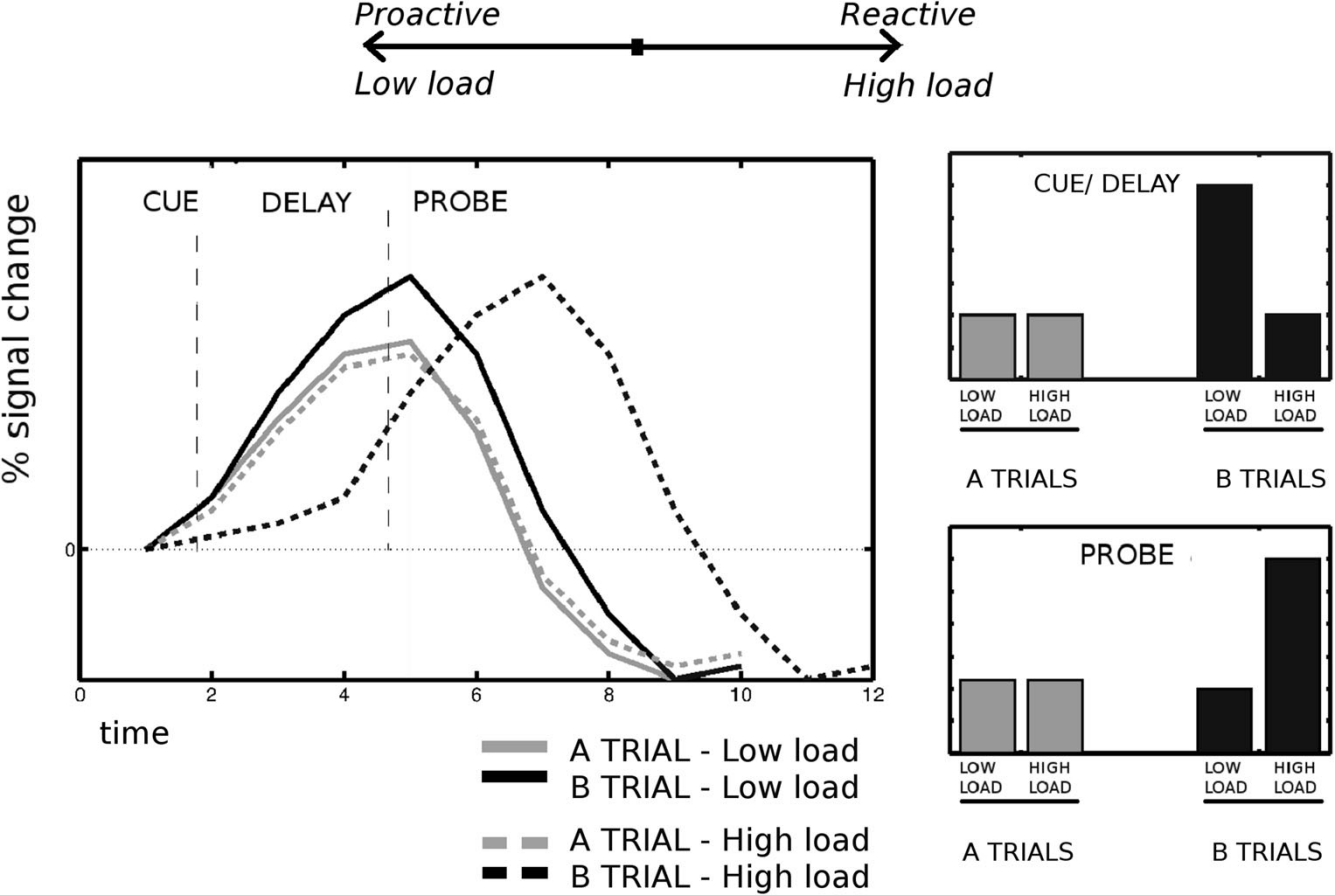
Hypothesized effects in the present study. The left panel displays the hypothesized BOLD responses for the different events, trial types, and levels of task context load in the regions involved in cognitive control. The BOLD responses were drawn based on results published in Perlstein et al. (2003), Edwards et al. (2010), and Braver et al. (2009). The right panels show the predicted effects of the context load manipulation for the hypothesized BOLD responses for each event type (i.e., A vs. B trials). We predicted that the activity would be larger after B than after A cues in the standard AX-CPT (low load; top right), consistent with the use of context. Furthermore, we expected that high task context load would induce reactive behavior, and therefore higher activity in B than in A trials in the probe period (bottom right)

## Materials and method

### The AX-CPT and manipulation of task context load

Each trial of the standard AX-CPT consists of two displays: First, a contextual cue is presented; then, after a delay, the probe is presented, and participants decide on whether or not the probe is a target and respond by pressing the appropriate button. In a target trial, the cue is typically the letter A, and the probe is the letter X; thus, the participants should respond to the target cue–probe pair (A followed by X) with a target response. For all other pairs (non-A and/or non-X), a nontarget response should be given. The AX target pair is typically presented in the majority of trials (e.g., 60%–70% of the trials), while the other three trial types are relatively infrequent (e.g., 6%–12% each; MacDonald, 2008; Servan-Schreiber et al., 1996). This creates an expectation for an X probe whenever the A cue is presented, and consequently a bias toward a target response. However, although the A cue predicts the target probe X with high probability, a non-X is presented in a minority of trials. In the trials with a cue other than A (generically called B, but that varied trial by trial), the following probe cannot be a target, and the cue can therefore be used to characterize the probe as a nontarget even before it is presented, so a nontarget response can be prepared. Proactive behavior is characterized by errors and slower reaction times when a non-X follows an A, presumably because participants expect an X and have to inhibit the prepotent target response. Reactive behavior is characterized by errors in trials in which a non-A is followed by an X (i.e., a BX trial), because participants react to the X without being able to use the context given by the B cue.

**Fig. 2:**
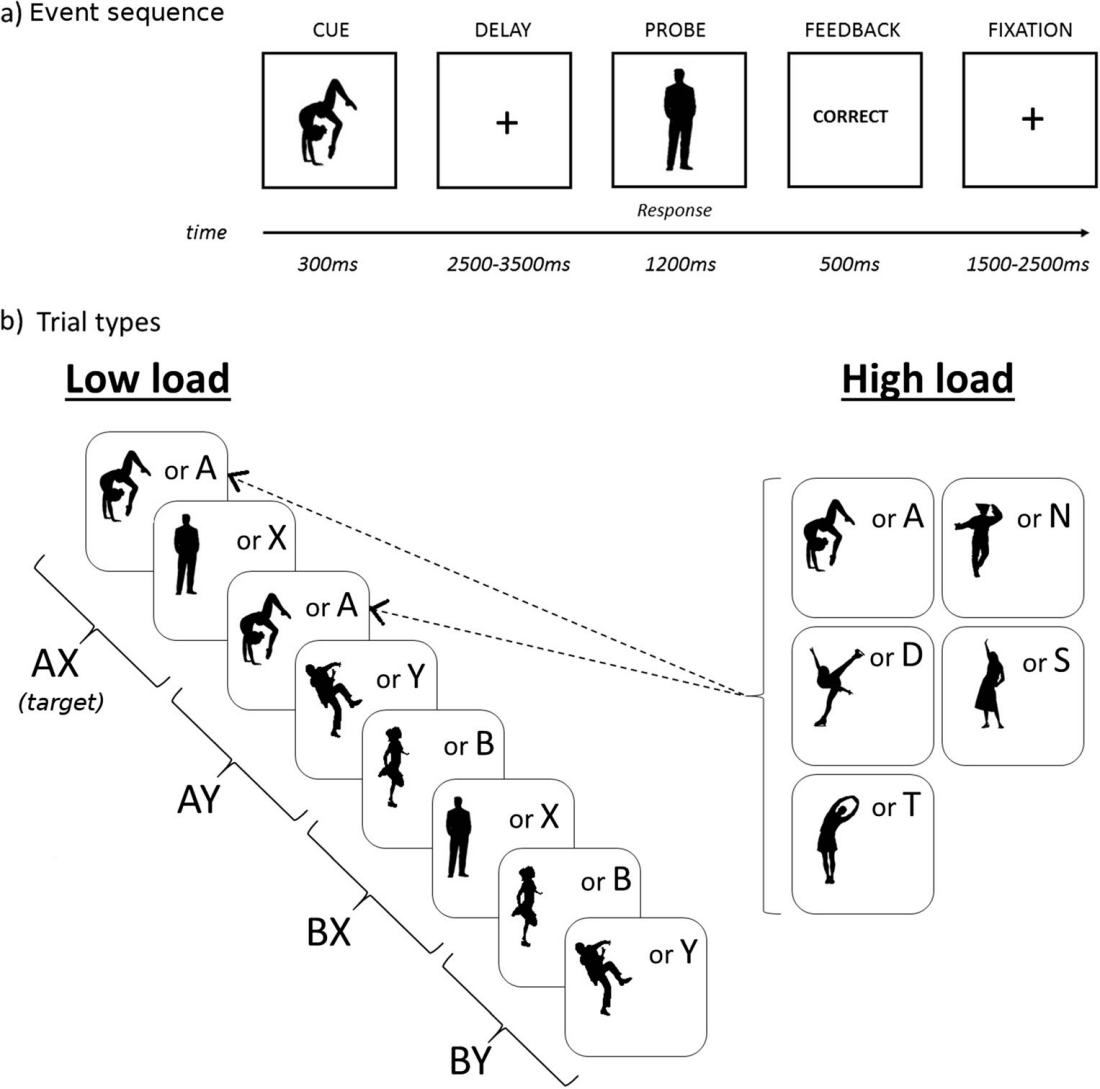
(**a**) Example of a trial in the fMRI experiment. (**b**) Left panel: Trial types and example of the stimuli in the low-load condition of the AX-CPT paradigm. In the target trial, the letter A (web) or a silhouette (fMRI) was presented as the cue or context, and the letter X (web) or a silhouette (fMRI) was presented as the probe. In the AX trials, participants responded to the target probe (X) by pressing the designated target button. In all other trials (AY, BX, and BY), participants responded to any probe by pressing the designated nontarget button. The B and Y stimuli actually varied across trials and were randomly selected from a set of 16 letters (web) or images (fMRI) different from the target (A and X) stimuli. Right panel: The high-load variant was identical to the low-load variant, with the exception that the A stimuli could be any of five different letters (web) or silhouettes (fMRI); each of the five stimuli was presented with equal probability. The frequency of trial types was identical across load conditions

In the present study, we aimed to test whether a high task context load would induce a more reactive processing mode, as reflected in a relative increase in the BX trial error rate, and if so, whether the induced change in processing mode would also be reflected in temporal changes in cortical and brain stem BOLD activity. To this end, we manipulated task context load by increasing the number of A cues—that is, the number of cues that defined the context of a target trial. In the high-load version, the target pairs were AX, NX, DX, SX, and TX, with similar frequencies of each (one fifth of the total amount of target trials). Thus, the high-context-load condition required that participants maintained a larger set of task rules in WM while doing the task. An X was the only possible target probe, and other probes would be nontarget probes (generically called Y, but that varied trial by trial). In this way, the trial types that constituted the high-context-load variant were the same and were in the same proportion as in the low-load variant. For a more detailed description and illustration of the task, see Fig. 2. We first tested whether increasing load induced reactive behavior, by using letter stimuli in web-based experiments. Subsequently, we adapted the task for fMRI and ran a separate experiment in the MRI scanner.

### Web-based experiments

#### Participants

A total of 149 participants were recruited through CrowdFlower® (www.crowdflower.com; mean age 32.56 ± 9.75 years, age range 19–82; 98 male, 51 female). Each experiment took about 15 min to complete, and participants were paid 0.80 USD for their effort.

#### Design and procedure

Due to limitations in the available effective testing time for web experiments, we chose a between-subjects design in which each participant was tested on one of three AX-CPT context load conditions [either low (*N* = 47), medium (*N* = 50), or high load (*N* = 52)]. For the web version of the AX-CPT, we used letters, as is illustrated in Fig. 2. The total number of trials was 150; of these, 70% were AX trials. The proportions of all other trial types (AY, BX, BY) were 10% each. The low-load condition was similar to a standard AX-CPT. The letter A, and only the letter A, was the target cue (i.e., target pair AX). In the experiment with medium context load, the target cues were the letters A, N, and D (yielding target pairs AX, NX, and DX), and in the high-context-load experiment, the target cues were A, N, D, T, and S. In all of the load conditions, the X was the only target probe, as in the standard version. The letters [B, C, E, F, H, I, J, K, L, M, O, P, R, S, U] served as non-A and non-X stimuli (i.e., as B cues and Y probes). Participants received written instructions, including examples of the different trial types. They were instructed to press the “M” key on their keyboards for target trials and the “Z” key for nontarget trials. Prior to starting the actual experiment, they were required to complete ten trials of training with an accuracy of at least 80% in order to be able to proceed to the task. In each trial, the cue was presented for 500 ms, followed by a delay of 1,500 ms. The probe and the visual feedback (“Correct” or “Wrong”) were each presented for 700 ms. The intertrial interval was 1,000 ms. The experiments were coded in JavaScript.

### fMRI experiment

#### Participants

On the basis of the effect sizes calculated from the web-based experiment, we expected the differences in accuracy between trial types in the low- and high-load conditions (in particular on BX trials, which would index reactive behavior) to be of medium effect sizes (*η*^2^ ~ .07). The sample size for the corresponding effect size and a power of .95 was estimated to be at least *N* = 24. Thirty-one adults (20 females, 11 males) were recruited through social media to participate in the study (mean age 27 ± 6 years, range 18 to 40). Before starting the experimental part, participants were asked to respond to a brief questionnaire to test whether they satisfied the inclusion criteria (no serious neurological or psychiatric illnesses, no vision or language problems). Participants gave their informed consent and were paid with a gift card equivalent to 200 Norwegian kroner (NOK) to complete the session, which took approximately 2 h. The study was conducted in accordance with the Declaration of Helsinki and with institutional guidelines, and was approved by the local ethics committee.

#### Design and procedure

To be able to run a within-subjects design in the MRI scanner, we made some adjustments to the paradigm. The fMRI experiment included two levels of context load (i.e., low and high) and consisted of 200 trials in total. We also increased the proportions of AY, BX, and BY trials to 12% each. The remaining 64% of trials were AX trials. We employed black silhouettes of people on a white background as stimuli, as is illustrated in Fig. 2. We did this mainly to avoid language familiarity influences. The silhouettes could be easily differentiated from each other. Previous studies had shown highly consistent results across stimulus types in the AX-CPT task (Chatham et al., 2009; Jones, Sponheim, & MacDonald, 2010; Lucenet & Blaye, 2014; Paxton et al., 2007). In addition, we expected that the silhouettes might be more engaging than overlearned letters. The task was coded in Psychtoolbox (Brainard, 1997). In the low-context-load condition, one particular silhouette was designated as the target context (similar to A in the letter version), and a different silhouette was the target probe (similar to X in the letter version), as is illustrated in Fig. 2. In the high-load condition, five silhouettes acted as the target context cues (see Fig. 2, right panel). To avoid item-specific effects, the whole set of silhouettes varied across participants, such that the target silhouettes for one participant were distractor silhouettes for another participant.

Each trial consisted of presentation of the cue for 300 ms, followed by a jittered delay period (mean duration 3,000 ms, range 2,500–3,500 ms). Then the probe was presented for 1,200 ms, and participants had to generate a response. Visual feedback was presented for 500 ms. Trials were separated by fixation intervals (mean duration 2,000 ms, range 1,500–2,500 ms). These parameters were based on the design used by recent work employing the AX-CPT tasks and fMRI (Lopez-Garcia et al., 2016). The participants received written and oral instructions and completed practice trials on a computer (ten trials per level of load) outside the scanner. Afterward, the full experiment was run in the scanner.

### Analysis of behavioral data

Accuracy and the mean reaction times (RTs) from correct trials were estimated for each trial type (AX, AY, BX, BY) in all experiments. For the web-based experiment, nonparametric tests were used to analyze accuracy, since the data were not normally distributed (Kolmogorov–Smirnov test for normality: *p*s < .001 for all variables). Friedman’s test was applied in order to obtain the main effect of trial type for each experiment, and the Wilcoxon test was used for post-hoc paired tests. Context-*d'* is based on signal detection theory (Stanislaw & Todorov, 1999) and is a measure of the ability of an individual to differentiate targets from distractors. It was estimated on the basis of hits in AX trials and false alarms in BX trials (Gonthier, Macnamara, Chow, Conway, & Braver, 2016). Total accuracy and context-*d'* were compared across experiments by means of *U* tests. The effect size for nonparametric tests was measured with *η*^2^ using an open-source tool by Psychometrica (www.psychometrica.de/effect_size.html). Differences in RT distributions were tested on correct trials with repeated measures analysis of variance (ANOVA), with trial type as within-subjects factor and experiment (i.e., load level) as a between-subjects factor. Effect size was measured with generalized *η*^2^ (*η*^2^_G_). This measure is preferable for repeated measures ANOVA, as compared to either *η*^2^ or partial *η*^2^, since it allows for comparison of different studies (Bakeman, 2005; calculator available at https://github.com/VeronicaMaki-Marttunen/Generalized-eta-square.git). All the data were analyzed with IBM SPSS.

For the in-scanner behavioral data, accuracy was analyzed using nonparametric tests, because it was nonnormally distributed (Kolmogorov–Smirnov test for normality: *p* < .001). Friedman’s test was applied in order to obtain the main effect of trial type, and the Wilcoxon test was used for post-hoc paired tests. For RTs, a repeated measures ANOVA was used, correcting for nonsphericity. Only correct trials were included in the RT analysis. In addition, we checked whether the sequence in which the participants encountered the different load levels (high load first or low load first) had an effect on accuracy in the different trial types. We found no significant effect of sequence [main effect: *F*(1, 29) = 0.21, *p* = .65; interaction of sequence by trial type: *F*(3, 87) = 0.62, *p* = .62].

A separate analysis was conducted to examine whether individual differences in the trend toward more or less proactive behavior, were reflected in different activity patterns. Participants were categorized according to their response pattern in the standard AX-CPT (low load) as proactive, reactive, or intermediate (as in Mäki-Marttunen et al., 2018). For this purpose, we calculated for each participant a proactive behavioral index (PBI). The PBI, based on BX and AY errors, was created to classify proactive versus reactive control modes. The PBI was calculated as in previous works: (AY – BX)/(AY + BX), where AY is the error rate in AY trials and BX is the error rate in BX trials (Braver et al., 2009; Gonthier et al., 2016). When the error rate was 0 for both types of trials, the PBI was set to 0. After calculating the PBI, the proactive group (PBI > 0) included *N* = 9 participants, the intermediate group (PBI = 0) had *N* = 13, and the reactive group (PBI < 0) had *N* = 9. We also estimated the context-*d'* for the fMRI sample.

### MRI acquisition and analysis

Participants were scanned in a 3-T Philips MRI scanner at Rikshospitalet, Oslo. Each scanning session started with an anatomical scan. Afterward, four runs of functional images were acquired while subjects performed the AX-CPT task. Whole-brain functional images were acquired using a spin-echo echoplanar (EPI) sequence sensitive to blood-oxygen-level-dependent (BOLD) magnetic susceptibility (TR = 2,208 ms; flip angle = 90°; number of slices: 42; voxel size: 3 mm^3^). Each functional run lasted about 6 min, and in each run 225 volumes were collected. Finally, a neuromelanin-sensitive scan was acquired, with the objective of further improving the localization of the LC. The slices for this scan were set in order to cover the brain stem (T1–TSE, TR: 600 ms, TE: 14 ms, voxel dimension: 0.4 × 0.49 × 3 mm, flip angle = 90°; number of slices: 10). This scan lasted 12 min.

The stimuli were projected onto a screen positioned at the head end of the scanner. Participants viewed the screen through a mirror placed on the head coil. Responses were given via buttons on an fMRI-compatible joystick device, with one button corresponding to the target response (AX) and another to the nontarget response. Each fMRI session consisted of two runs for each load level in a semi-random order across participants. Before each run, a screen informed the participants of the pair of targets they had to identify during the run. Each run consisted of two task blocks of 25 trials each. In total, there were 100 trials per load level. Each run started with a task block, followed by 20 s of fixation, then another task block, and finally another 20 s of fixation. The different trial types (AX, AY, BX, BY) were presented in semi-random order within task blocks.

The functional images of each participant were first visually inspected for anomalies and then were submitted to a standard preprocessing pipeline, using SPM 12 implemented on MATLAB. The images were first corrected for time delays and realigned using six parameters of movement. The data were normalized to a standard template and smoothed (8-mm full-width-at-half-maximum [FWHM] Gaussian kernel). For the analysis of activity in the LC, a smaller Gaussian kernel was used (3-mm FWHM). Additional motion correction was performed by scrubbing the volumes with excessive movement by using FSL functions.

The preprocessed images were submitted to a first-level analysis. Event-related activation was estimated with a general linear model (GLM) in which only correct trials were included. Cue/delay and probe types were modeled as separate events with a canonical hemodynamic response function plus time derivatives basis functions. The modeled events were cue type (A, B) and probe type (X of AX, X of BX, Y of AY, Y of BY) for each level of load (6 × 2 = 12 events). A high-pass filter (cutoff: 250 s) was applied in order to remove signal drift. Contrasts were generated for each cue and probe type, collapsed across load levels and for each load level. The parameter estimates from each participant’s GLM were submitted to second-level tests treating participants as a random factor in *t* tests and ANOVA.

To study the effect of load in the activity associated with the different cue and probe types, a full-factorial analysis was performed, with cue type and load as factors. To obtain the neural correlates of the different cue and probe types, we performed a small-volume correction (SVC) in a mask defined on fronto-parietal regions known to be involved in cognitive control. The mask for the SVC was derived from a meta-analysis of previous studies using the AX-CPT and fMRI; it consisted of the merge of the different areas (see Fig. 4 in the Results section). This approach ensures that the activation found is related to the task, and therefore it controls for Type I errors; such an approach had previously been used in fMRI AX-CPT studies to assess the effect of manipulations and/or to compare etiological groups (Edwards et al., 2010; Locke & Braver, 2008). On the other hand, it allowed for the use of more lenient cluster-forming thresholds that would allow for improved sensitivity/power and protected against Type II errors. The parameter estimates in Fig. 6 below were extracted from a 5-mm sphere defined around the coordinate of maximum effect size in the region indicated, to show the direction of the effects (no statistical calculations were performed).

Given our interest in the brain stem, we performed a region-of-interest (ROI) analysis of activity in the VTA/SN and LC. For the VTA/SN, we used a combined VTA/SN mask derived from probabilistic atlases of the structures (Murty et al., 2014). The VTA and SN masks partially overlap and therefore we combined them; the resulting mask was cropped to be spatially restricted to the brain stem. For the LC, given its small size and the variability of its location in the brain stem across individuals, we defined individual masks in the high-resolution T1–TSE images and used them for an ROI analysis. The LC of each individual was delineated on the axial slices of the neuromelanin scans. The position of the nuclei was determined in the pons as the voxels of hyperintensity on either side of the fourth ventricle (an example is displayed in Fig. 5 below), following the procedure employed in previous studies (Krebs et al., 2018). The T1–TSE scan of each individual was co-registered to the corresponding structural image, the structural was co-registered to the mean functional and then the deformation field calculated during the normalization step was applied to the co-registered T1–TSE image. The final size of the individual unilateral masks of LC was, on average, eight voxels in the normalized space. For the ROI analysis, the data was extracted from the contrast images with rfxplot toolbox (Gläscher, 2009). The parameter estimates of each condition and load level were extracted within the individual masks in the voxel of maximum effect size of the independent contrasts (cue A, cue B, etc.). Then we performed repeated measures ANOVA applying load (levels: low and high) and cue type (A, B) or probe type (X of AX trials, Y of AY trials, X of BX trials, and Y of BY trials) as within-subjects factors, depending on the events examined. For the comparison of groups, we included group as a between-subjects factor. For the comparison of activity in the right dlPFC across groups, we first obtained the voxels within the right dlPFC mask of the meta-analysis that were active in the B > A contrast (*p* = .001), and then we extracted data from a 5-mm sphere around the individual coordinates with the maximum B > A effect size.

### AX-CPT meta-analysis

To identify the regions related to cognitive control as studied by the AX-CPT, we performed a meta-analysis based on previous work using the AX-CPT and fMRI (Edwards et al., 2010; Lesh et al., 2013; Lopez-Garcia et al., 2016; Paxton et al., 2007; Perlstein, Dixit, Carter, Noll, & Cohen, 2003; Poppe et al., 2016; Poppe, Carter, Minzenberg, & MacDonald, 2015; Yoon et al., 2008). The list of coordinates can be found in Table S5. We extracted the coordinates reported for the B > A contrast, transformed them from Talairach space into MNI space, and fed a meta-analysis. We used the GingerALE tool 2.3.6 (www.brainmap.org; Eickhoff, Bzdok, Laird, Kurth, & Fox, 2012; Eickhoff et al., 2009) and the following settings for a single-study meta-analysis: minimum volume: 10 mm^3^, mask size: less conservative, method: Turkeltaub, and additional FWHM: 10 mm. The meta-analysis gives a parametric map of the probabilities of each voxel being activated in each study. We then created a mask by applying a threshold of .5 (see Fig. 4 below for the resulting map of ROIs). The mask is available upon request.

## Results

### Behavior: Task context load induces reactive shifts of cognitive control

#### Web-based experiments

**Fig. 3:**
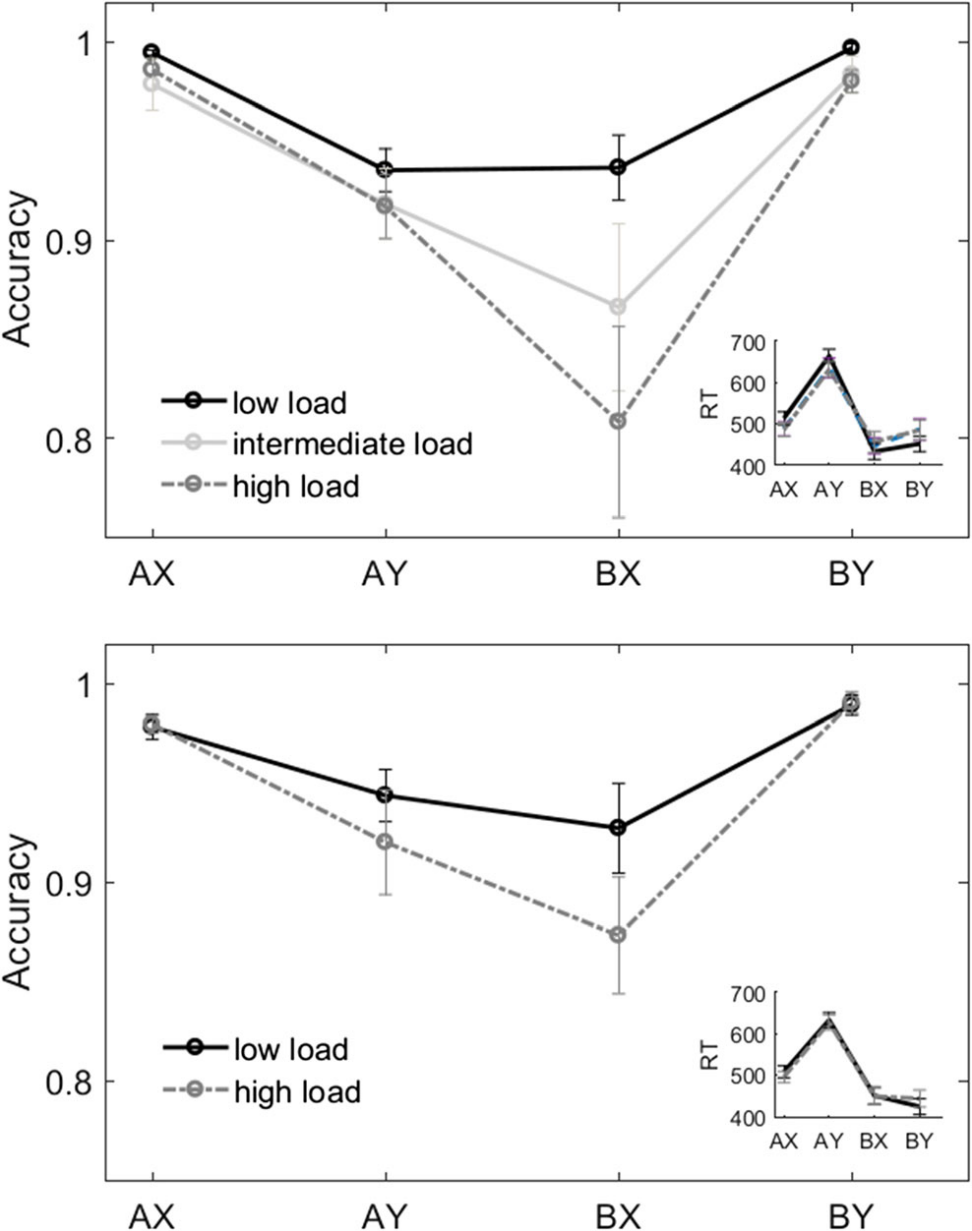
Behavioral results. (Top) Web-based experiment. (Bottom) fMRI experiment. In each panel, the main plot indicates accuracy per trial type, and the inset shows reaction times (RTs). Error bars indicate standard errors of the means. Higher load induced more reactive behavior, characterized by reduced accuracy on BX trials

The results obtained in the web-based experiments are depicted in Fig. 3, top panel. Average accuracy was higher with low load than with either intermediate or high load (average accuracy: low load, 0.96 ± 0.03; intermediate load, 0.90 ± 0.12; high load, 0.91 ± 0.09; *U* test: low vs. intermediate, *Z* = 2.42, *p* = .015, *η*^2^ = .06; low vs. high, *Z* = 3.09, *p* = .002, *η*^2^ = .094). We found a main effect of condition on accuracy at all levels of load (Friedman test, main effect of condition: *p* < .001), with more errors in AY than in AX and BY trials (Wilcoxon test, *p* < .001) and more errors in BX than in AX and BY trials (Wilcoxon test, *p* < .001). Under high load, participants committed more errors in BX than in AY trials (Wilcoxon test, *Z* = 2.71, *p* = .007, *η*^2^ = .14), but they did not under low or intermediate load (*p* = .478 and *p* = .242, respectively). When comparing between load levels, accuracy was lower under high than under low load for AX, BX, and BY trials (*U* test, *Z* = 2.73, *p* = .006, *η*^2^ = .075; *Z* = 2.79, *p* = .005, *η*^2^ = .080; and *Z* = 2.62, *p* = .009, *η*^2^ = .070, respectively). Regarding RTs, AY trials were significantly slower than all other trials [trial type effect: *F*(3, 417) = 38.35, *p* < .001, *η*_G_^2^ = .061]. There was no significant load effect (see Fig. 3).

The context-*d'* was significantly lower under high than under low load (context-*d'*: high load, 3.98; low load, 5.07; *U* test: *Z* = 3.08, *p* = .002, *η*^2^ = .096). Context-*d'* was lower under intermediate than under low load, although the difference did not reach statistical significance (context-*d'* for intermediate load: 4.30; *U* test, *p* = .080, *η*^2^ = .191).

Given the wide age range of the participants in the web-based experiments, we repeated the analysis after restricting the sample to young adults (19–50 years). The results are similar to those obtained with the full age range and are reported as supplementary data (Appendix A).

In summary, the load manipulation made the task more difficult in general, as indicated by the decreased context-*d'*, but also made the strategy more reactive, as indicated by the relative increase in the BX error rate. In the following experiment, we tested whether this finding could be replicated in a within-subjects design carried out in a controlled setting in the MRI scanner.

#### In-scanner behavioral results

The behavioral results obtained in the fMRI experiment are depicted in Fig. 3, bottom panel. Mean total accuracy was nominally higher under low than under high load (low load, 0.96 ± 0.04; high load, 0.94 ± 0.06), but the difference did not reach statistical significance (*U* test: *Z* = 1.91, *p* = .056, *η*^2^ = .118; Table S2). Statistical tests showed similar patterns of error rates for the two levels of load: Participants made more errors in AY and BX than in AX and BY trials (Friedman test: trial type effect in low load, *χ*^2^ = 12.68, *p* = .005; high load, *χ*^2^ = 24.41, *p* < .001; Fig. 3, bottom). BX accuracy was significantly lower for high than for low load (Wilcoxon test, *Z* = 2.17, *p* = .030, *η*^2^ = .152). AX, AY, and BY errors did not significantly differ between levels of load (*p* > .5).

When looking at the RTs, we found slower responses in AY trials than in the other trial types [trial type effect: *F*(3, 30) = 97.53, *p* < .001, *η*_G_^2^ = .07; Fig. 3, bottom]. There was no load effect on RTs. The context-*d'* decreased with load (low load, 3.76 ± 0.91; high load, 3.43 ± 1.01) although the difference did not reach statistical significance (*Z* = 1.76, *p* = .077, *η*^2^ = .1; Table S2). Further analyses provided additional evidence for a qualitative shift from proactive to reactive control with load in both the web-based and fMRI experiments (supplementary material, Appendixes B and C).

In general, the behavioral results in the fMRI experiment replicated those observed in the web-based experiment. Both experiments showed decreased accuracy and decreased context sensitivity (context-*d'*) with load, although the effect was significant only in the web-based experiment. Most importantly, we observed a significantly increased error rate in BX during high load as compared to low load in both experiments. The behavioral effects of the load manipulation were specific for accuracy and did not affect the distribution of RTs. Taken together, the results suggest that the load manipulation imposed higher WM demands that induced a shift in the allocation of cognitive resources into a more reactive strategy.

### Imaging results

#### Cognitive control network and brain stem regions show larger B-related than A-related activity

**Fig. 4:**
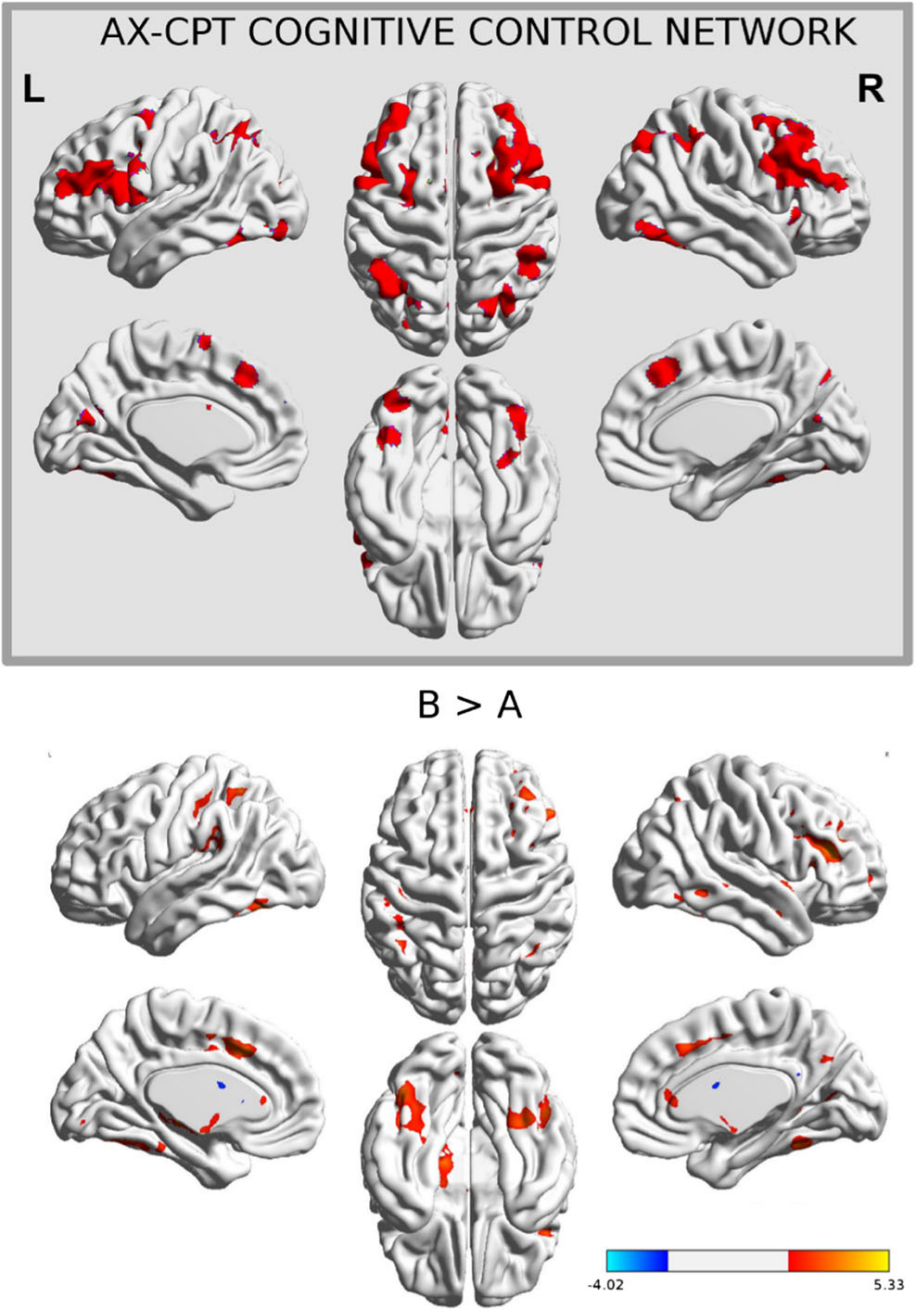
(Top) AX-CPT cognitive control network obtained from a meta-analysis. (Bottom) Main effect of cue type during the cue/delay period

We first defined the set of regions commonly reported in the literature to show larger activity during B than during A cues (B > A) by performing a meta-analysis of the previously published AX-CPT fMRI studies (Fig. 4). The resulting regions comprise bilateral inferior frontal gyrus, right middle frontal gyrus, bilateral inferior parietal lobe, supplementary motor area, and several clusters in the occipital cortex and bilateral cerebellum. The regions that were significantly different in the B > A contrast have been interpreted in the previous studies as the network underlying WM maintenance of the context provided by the B cues and/or the preparatory activity of the nontarget response.

We then analyzed the B > A differences in cue-related activity across levels of load in the present data set. In agreement with the meta-analysis results, B cues triggered larger activity than A cues in the supplementary motor area, the right middle frontal gyrus, and the left occipital and inferior parietal cortices (Fig. 4; the clusters are reported in Table 1). The contrast A > B did not show any significant activation. When looking at the probe-related brain activity, we found a significant effect of probe type in several fronto-parietal regions (Table S7).

**Table 1:** Cue-related activity: B > A

	Region	BA	Size	Coordinates			
				*x*	*y*	*z*	*Z*
RH	MFG	46	171	42	34	20	3.70
RH	IFG	45		54	30	14	3.51
RH	MFG	6	92	32	16	40	3.37
RH	IFG		12	46	22	12	3.00
LH	SMA		46	– 6	20	42	3.01
LH	IPL	40	75	– 38	– 52	56	3.26
LH	Cerebellum		42	– 44	– 66	– 16	3.19
RH	Cerebellum		135	30	– 46	– 28	3.19

RH, right hemisphere; LH, left hemisphere; MFG, middle frontal gyrus; IFG, inferior frontal gyrus; SMA, supplementary motor area; IPL, inferior parietal lobe. Clusters surviving small-volume correction on the mask obtained from the meta-analysis, peak level: *p* < .005, uncorrected. Cluster size is given in voxels.

**Fig. 5:**
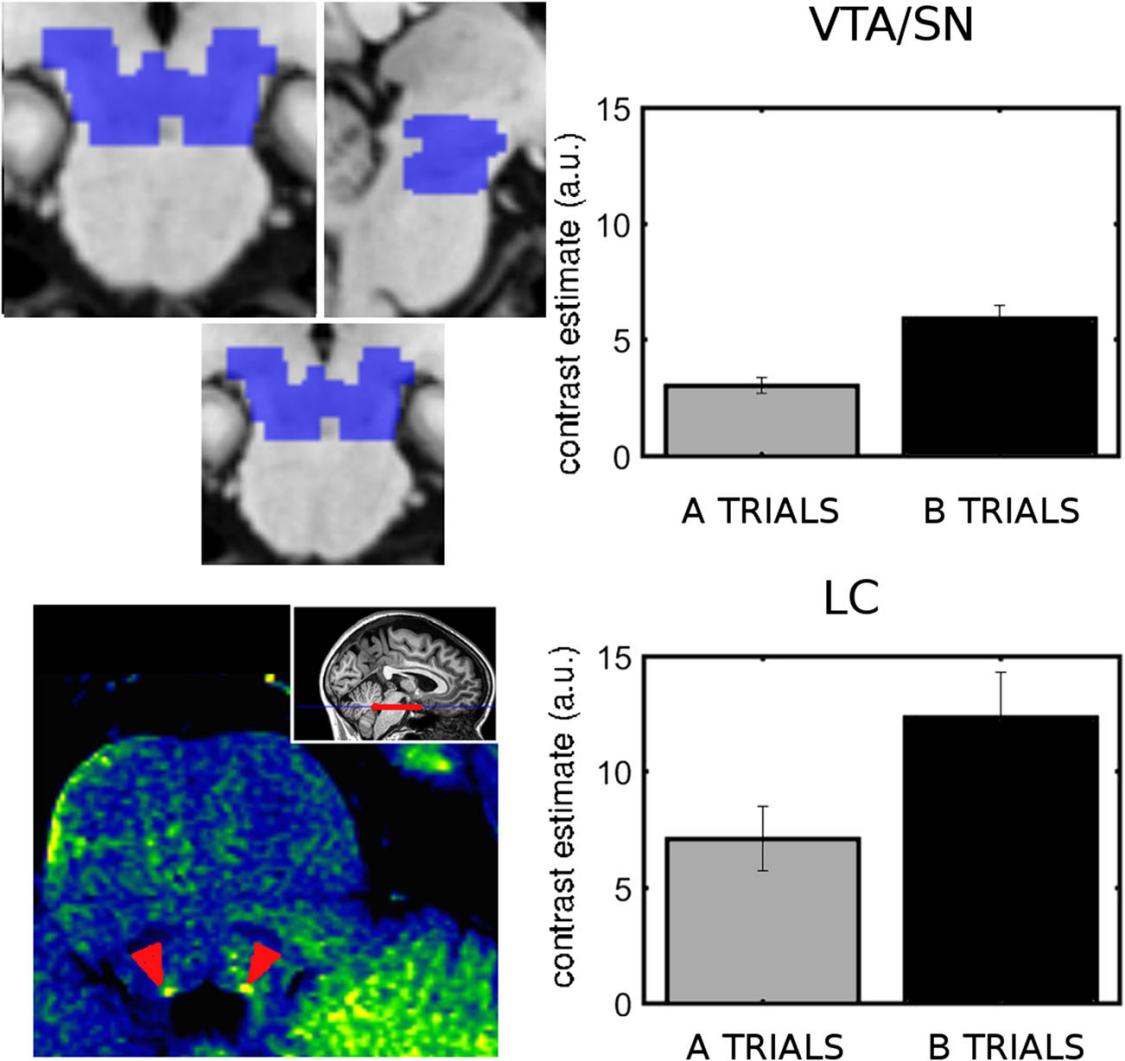
Effects of cue type in brain stem nuclei. (Top row) Ventral tegmental area/substantia nigra (VTA/SN). (Bottom row) LC. (Top left) Atlas-based mask of the VTA/SN (blue). (Bottom left) Example of localization of the LC on the neuromelanin scan of one individual. (Right column) Cue-related activity after the different cue types from the ROI analysis. Brain stem nuclei showed greater activity to contextual B cues than to A cues. Error bars indicate standard errors of the means

An important motivation of the present study was to investigate the activity of brain stem nuclei in cognitive control; therefore, we performed ROI analyses. In the VTA/SN, we found that B cues triggered greater activity than A cues [*F*(1, 30) = 21.86, *p* < .001, *η*^2^_G_ = .16]. Similarly, in LC, B cues triggered significantly greater activity than A cues [cue type effect: *F*(1, 30) = 8.18, *p* = .008, *η*_G_^2^ = .05; Fig. 5].

#### Context load reduces cue-related activity and increases probe-related activity in B trials in the right dlPFC

**Fig. 6:**
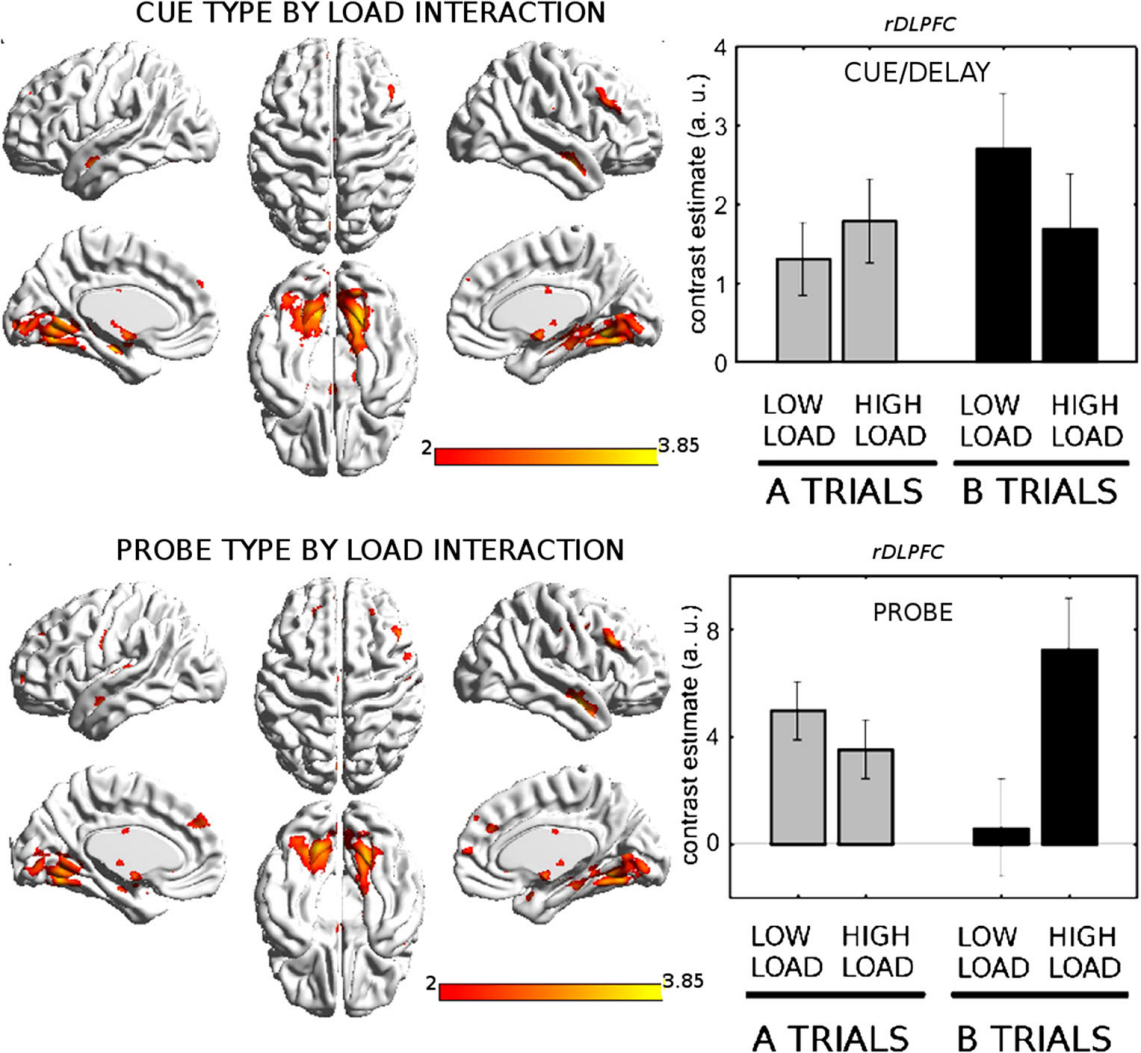
Brain maps corresponding to the cue type by load interaction (top) and probe type by load interaction (bottom). Right column: Activity obtained in 5-mm spheres in the right dorsolateral prefrontal cortex (dlPFC), coordinates *x* = 52, *y* = 20, *z* = 32 (cue by load interaction; Table S6) and *x* = 48, *y* = 22, *z* = 32 (probe by load interaction; Table S7). Higher load was related to reduced activity after B cues and increased activity for the probes of B trials in the right dorsolateral prefrontal cortex, in agreement with what was hypothesized (Fig. 1). Error bars indicate standard errors of the means

We next assessed the effect of the task-context load manipulation on brain activity. We found that B cues evoked greater activity at low than at high load in right middle frontal gyrus, right precentral area, and middle temporal lobe (Fig. 6 top, Table S6). When looking at the probe-related activity, we found that load modulated activity in B trials in the right middle frontal gyrus and precentral area, where high load evoked greater activity than low load (Fig. 6, Table S7).

In the brain stem, activity in VTA/SN showed no cue type by load interaction. In the probe period, the VTA/SN showed a significant probe type effect [*F*(1, 30) = 12.22, *p* < .001, *η*^2^_G_ = .034], with greater activity in probes of B trials (BX and BY) than in probes of A trials (AX and AY) (AX vs. BX, *p* = .001; AY vs. BX, *p* = .015; AX vs. BY, *p* = .004; AY vs. BY, *p* = .015). However, there was no significant effect of load.

In the LC, the effect of load and the interaction of cue type and load were nonsignificant (*p* > .2). In the probe period, there was significant activation [*F*(1, 30) = 28.6, *p* < .001], but the effects of probe type and load were nonsignificant (*p* = .076 and .40).

In summary, increasing load induced a shift of activity from cue to probe in B trials in the right dorsolateral prefrontal cortex, in accordance with our hypothesis (Fig. 1). The shift from cue/delay to probe-related brain activity is hypothesized to reflect the shift from a proactive to a more reactive mode of cognitive control. Higher probe-related activity reflects selection of the response the moment it is required—that is, triggered by presentation of the probe. This effect was not observed in the brain stem nuclei.

#### Analysis of individual differences: Activity in the right dlPFC and LC is modulated by context load only in proactive participants

In an exploratory analysis, we divided the sample in subgroups according to the PBI scores of each participant in the standard AX-CPT (low load). We hypothesized that the different processing modes would be reflected in different patterns of right dlPFC, VTA/SN, and LC activity.

**Fig. 7:**
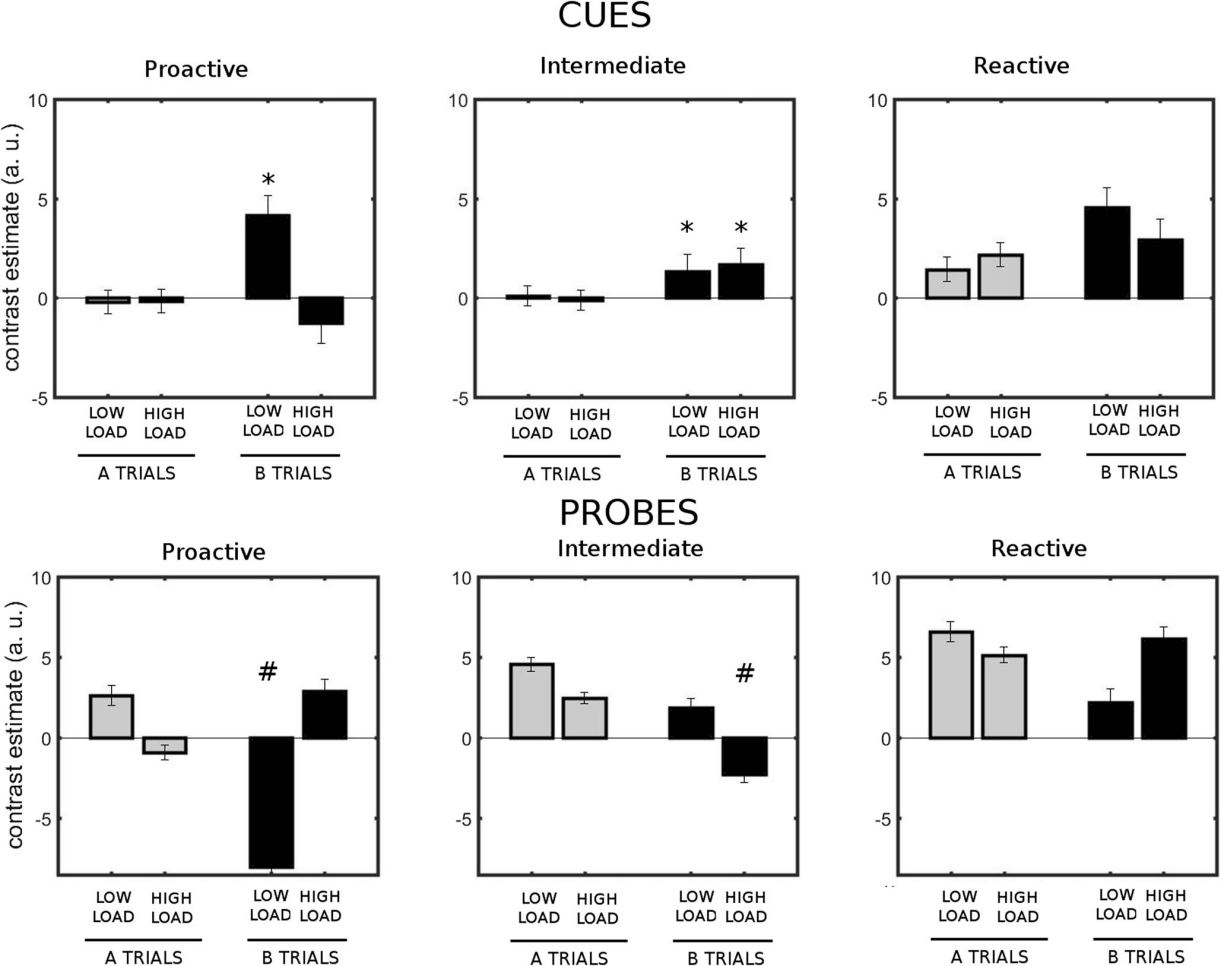
Cue-related (top row) and probe-related (bottom row) activity in the right dlPFC for both levels of load, in the groups of participants who behaved proactively, intermediately, and reactively under low load (left, central, and right columns, respectively). A shift of activity with increasing load was observed for B cues in the proactive but not in the intermediate or reactive group. ^*^Significant differences in cue-related activity between B and A trials in the corresponding load level. ^#^Significant differences in probe-related activity between B trials and A trials in the corresponding load level

We first investigated the activity patterns for the different groups in the active voxels of B > A within the right dlPFC (see the Method section). We performed a repeated measures ANOVA with cue type (A and B) and load level (low and high) as within-subjects factors and group as a between-subjects factor. The group effect was significant [*F*(2, 28) = 6.05, *p* = .007, *η*^2^_G_ = .12, Fig. 7], with the reactive group having larger activity than the other groups (reactive vs. proactive, *p* = .016; reactive vs. intermediate, *p* = .013). The Cue Type × Load × Group interaction was significant [*F*(2, 28) = 4.03, *p* = .029, *η*^2^_G_ = .05]. Planned comparisons showed that the paired contrast B versus A was significant in the proactive group only with low load (*p* = .002), and in the intermediate group with both levels of load (low, *p* = .017; high, *p* = .001). All other contrasts were nonsignificant. In the probe period, we found a group effect [*F*(2, 28) = 5.80, *p* = .008, *η*^2^_G_ = .13], with the reactive participants having greater activity than the proactive participants (*p* = .006). The Probe Type × Load × Group interaction was significant [*F*(2, 28) = 4.72, *p* = .017, *η*^2^_G_ = .07]. Planned comparisons showed that in the proactive group, the activity was significantly lower in B trials than in A trials, but only with low load (*p* = .004), and that there was greater activity in B trials with high as compared to low load (*p* = .043). In the intermediate group, there was lower activity in B trials with high as compared to low load (*p* = .001).

**Fig. 8:**
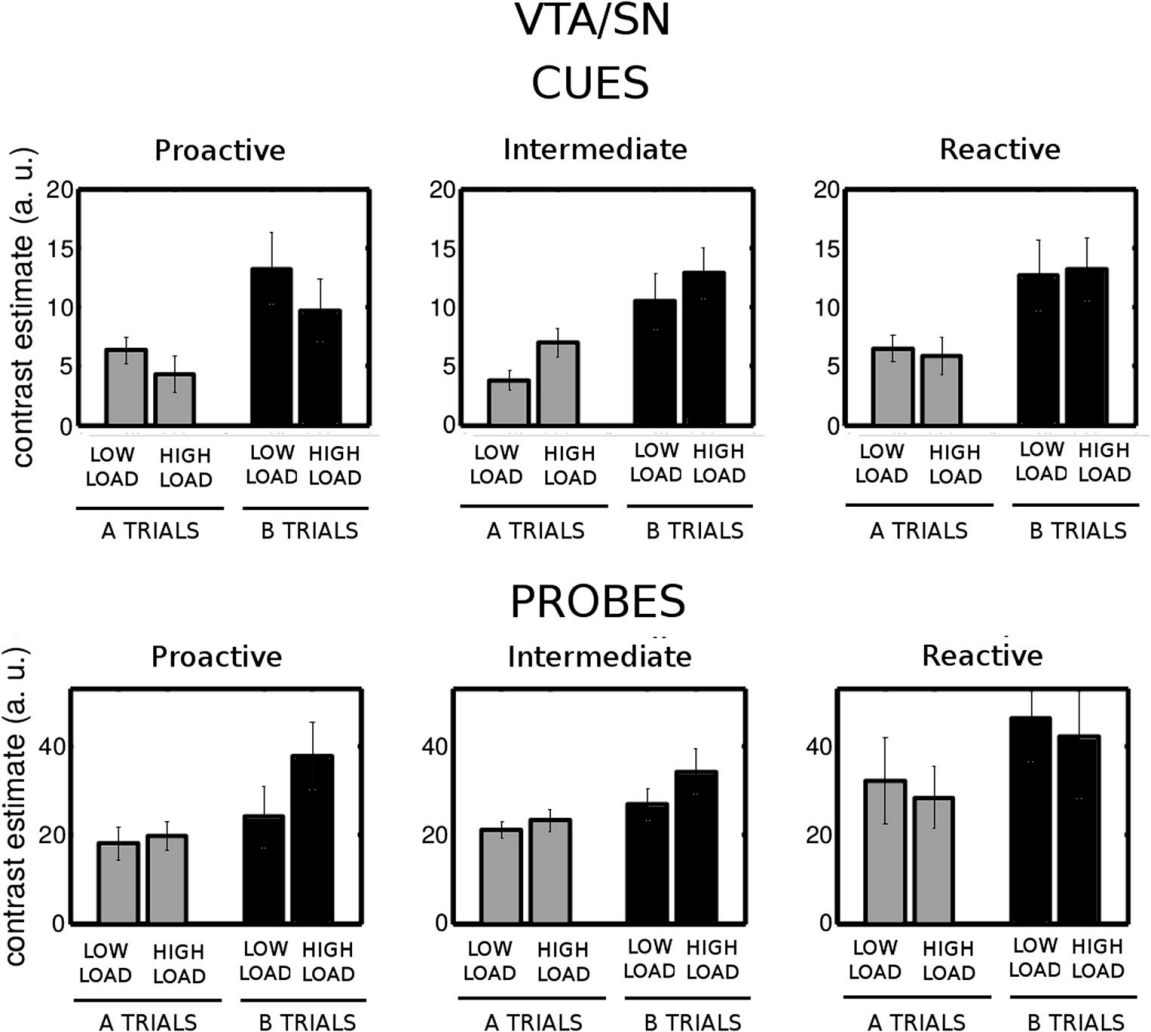
Cue-related (top row) and probe-related (bottom row) activity in the VTA/SN for both levels of load in the groups of participants who behaved proactively, intermediately, and reactively with low load (left, central, and right columns, respectively)

**Fig. 9:**
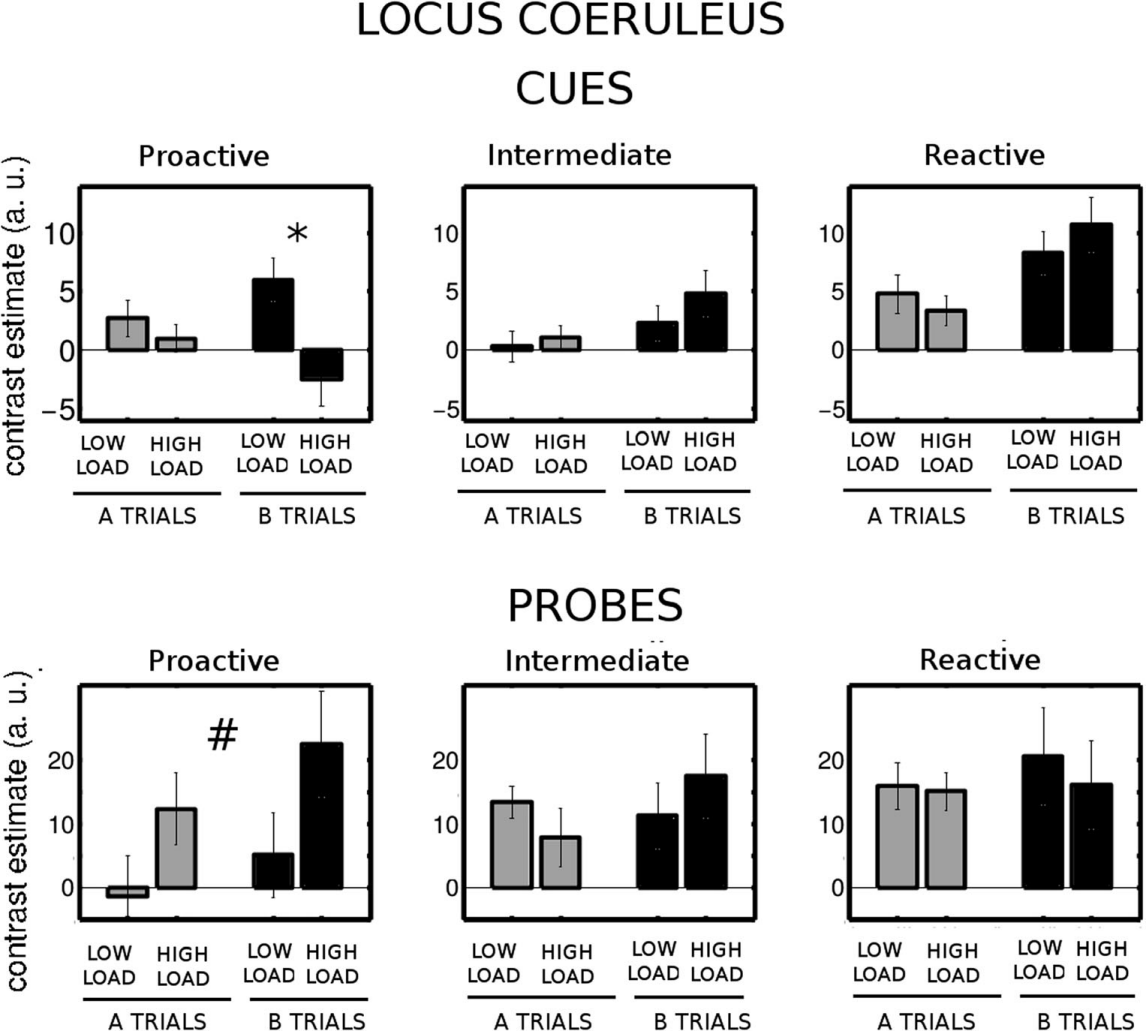
Cue-related (top row) and probe-related (bottom row) activity in the LC for both levels of load in the groups of participants who behaved proactively, intermediately, and reactively with low load (left, central, and right columns, respectively). A shift of activity with increasing load was observed for B cues in the proactive but not in the intermediate or reactive group. ^*^Significant difference between low and high load in B trials. ^#^Significant differences between proactive and reactive participants with low but not with high load

We then examined the brain stem nuclei. In the VTA/SN, we found no group effect or interactions (Fig. 8). In LC (Fig. 9), we found significant two-way interactions of Load × Group [*F*(2, 28) = 4.6, *p* = .019, *η*^2^_G_ = .059] and Cue Type × Group [*F*(2, 28) = 4.66, *p* = .018, *η*^2^_G_ = .031], as well as a significant three-way Load × Cue Type × Group interaction [*F*(2, 28) = 3.74, *p* = .036, *η*^2^_G_ = .033]. We further explored these interactions and found that reactive and intermediate participants had significantly greater B cue-related activity under high load than did proactive participants (reactive vs. proactive, *p* = .002; intermediate vs. proactive, *p* = .020). In addition, proactive participants had a significantly greater B cue-related activity with low than with high load (*p* = .025). This effect was not significant in the reactive and intermediate groups (*p* > .2).

We then analyzed the LC activity in the probe period. We found a significant Load × Group interaction effect [*F*(2, 28) = 3.58, *p* = .041, *η*^2^_G_ = .040]. Probe-related activity was greater in the reactive than in the proactive group for low load (*p* = .046), but not for high load. The comparison between low and high load in the proactive group pointed to an increase in probe-related activity with load, although it did not reach statistical significance (*p* = .083).

In summary, only the proactive group showed a modulation of right dlPFC and LC activity by context load, such that increased context load caused a reduction in B cue-related activity and an increase in probe-related activity, in line with what was expected in a region modulated by the load manipulation (Fig. 1). The activity in the VTA/SN presented no differential patterns with load across groups.

## Discussion

In the present study, we demonstrated that increased task context load in the AX-CPT induces a more reactive processing mode in healthy adults, and we also observed a reactive shift in brain activity. In particular, increased load induced an increased error rate in BX trials as well as a reduction in cue-related activity and an increase in probe-related activity in the right dlPFC. The analyses of brain stem activity revealed that both the VTA/SN and LC were involved in context updating in the cue period. Finally, an exploratory analysis of PBI-based subgroups of individuals revealed that task context load influenced right dlPFC and LC but not VTA/SN activity, and only in proactive individuals. Each of these findings and their potential implications are discussed below.

### The task manipulation and induced reactive behavior

Working memory enables maintenance of contextual information over time, but insufficient capacity to maintain relevant contexts may reduce the ability to guide thoughts and actions in accordance with goals. Working memory demands do not only limit the capacity of cognitive control, but may also change the strategy by which cognitive control is exerted. In particular, remembering and keeping track of multiple relevant contexts may influence the temporal dynamics of context updating. Motivated by dual-process accounts of cognition, such as the DMC and others (Braver, 2012; Braver et al., 2007; Engle & Kane, 2004; Evans & Stanovich, 2013; Jacoby et al., 1999; Stanovich & West, 2000), and by research on individual differences on the AX-CPT (Burgess & Braver, 2010; Redick, 2014; Unsworth & Engle, 2007), we hypothesized that when faced with changes in context load, the cognitive control strategy might be adaptively adjusted. In a novel AX-CPT variant, we manipulated the task context load in a way that allowed us to keep the trial type structure identical across load levels. The instructions and the frequency and sequencing of trials were the same across variants, providing a “tightly controlled contrast” to the standard AX-CPT (Locke & Braver, 2008).

The overall performance appeared to be somewhat compromised by high load, as indexed by nominally lower context-*d'* and overall accuracy, but these effects were small and did not reach statistical significance. This result suggests that performance under high context load was not characterized by a general WM undercapacity or deficient motivation in the sample. On the contrary, the context load manipulation successfully induced a more reactive control strategy in the participants, as evidenced by increased error rates in BX but not AY trials under high context load. The strategy shift was first demonstrated in the web-based experiment and was subsequently replicated in the scanner. The shift toward a more reactive control strategy might have been caused by different factors. For example, a proactive processing mode might have become too demanding to entertain in the high load condition given the WM resources available to the participants. That is, although the participant might have had sufficient WM capacity to successfully perform the task (i.e., differentiate between targets and distractors), the WM demands of the high-context-load condition might have enforced a strategy shift in order to maintain the performance level. An individual differences approach could be informative on this issue—for example, by comparing the degree of strategy shift between high versus low WMC. Although the present study did not include direct WMC estimates, several studies have shown that context-*d'* is positively related to WMC (Boudewyn et al., 2015; Redick & Engle, 2011; Richmond et al., 2015; Stawarczyk, Majerus, Catale, & D’Argembeau, 2014). The analysis of context-*d'* in our data revealed significantly higher scores for proactive than for reactive individuals, but we could not show significant differences between PBI groups on the behavioral effects of the load manipulation. This might be due to limited statistical power in the split group analysis and/or that context-*d'* does not fully capture the relevant elements in WMC. However, the exploratory analysis of cortical and brain stem activity revealed a reactive shift only in proactive participants, suggesting that limitations in cognitive capacity per se may not have been the factor driving the strategy shift.

An alternative explanation could be that high context load reduced the utility of the effort needed to sustain proactive mode, due to increased processing costs. The allocation of cognitive effort can be conceptualized as a value-based decision in which the costs and benefits associated with investing cognitive resources are weighted against each other (Kurzban, Duckworth, Kable, & Myers, 2013; Shenhav, Botvinick, & Cohen, 2013; Shenhav et al., 2017; Westbrook & Braver, 2016). In the present experiment, we did not have access to information on the computations involved in such an evaluation. However, since activity in the LC–norepinephrine (LC-NE) system can be used as an index of current engagement in a particular task, such activity can be understood as an outcome of this evaluation process (see below for a discussion on the involvement of the LC in context updating, and on individual differences in task-induced LC activity).

Another explanation could be that high A cue load conditions were associated with smaller repetition suppression effects, since each of the five stimuli that served as the A cue was presented less frequently than the single stimulus that served as the A cue in the low-load condition. However, each of the stimuli was presented multiple times during training and over the course of the experimental trials, as were all stimuli used in the task, suggesting that large differences in repetition suppression effects should not be expected. In line with this reasoning, inspection of Fig. 6 indicates that the cue type by load interaction was primarily driven by differences in the processing of B cues.

A substantial literature on the AX-CPT and cognitive control suggests that healthy young adults tend to behave proactively under standard task requirements. In contrast, young children behave reactively until they are 4–5 years old, after which they develop a more proactive response pattern (Chatham et al., 2009; Lucenet & Blaye, 2014). Also, older people and patients with dementia or schizophrenia present deficits in proactive control (Braver & Barch, 2002; Braver, Satpute, Rush, Racine, & Barch, 2005; Edwards et al., 2010; Lesh et al., 2013; Paxton et al., 2007; Poppe et al., 2015; Yoon et al., 2008), although they have no deficits in purely reactive control (Barch & Shefield, 2017; Bugg, 2014; Mann, Footer, Chung, Driscoll, & Barch, 2013; Van Gerven, Hurks, Bovend’Eerdt, & Adam, 2016).

Manipulations of task requirements have previously been used to induce within-subjects changes in control strategy in healthy, young adults. For example, Marklund and Persson (2012) showed that cues can be used to induce participants to employ a proactive processing strategy to overcome stimulus interference and improve overall task performance. Speer et al. (2003) manipulated the expectation of subsequent WM load and observed a more reactive strategy associated with high load expectation. Our context load version of the AX-CPT intended to simulate compromised WM capacity as seen in old people and patients with dementia or schizophrenia, by loading WM in healthy young individuals. Consistent with this idea, and with the notion of domain-generality described in DMC, the results of the present experiments seem to mimic the effects observed for groups of individuals with reduced WM capacity. These results suggest that the ability to process context information so as to guide the flexible adaptation of cognitive strategies to current demands is an important limitation underlying the performance deficits observed for children, old adults, and patients with neuropsychiatric diseases. Our findings complement previous studies of the AX-CPT in which reactive control was induced by introducing manipulations that altered the structure of the AX-CPT (such as the introduction of no-go trials, a secondary task, or perceptual interference; Braver et al., 2009; Gonthier et al., 2016; Lositsky, Wilson, Shvartsman, & Cohen, 2015). In line with previous work, we interpret this as a signature of adaptive flexibility; that is, participants stopped relying on the cue and started relying on the probe to select the response. We speculate that a weaker mental representation of the target cues, in addition to the control demand imposed by each trial, may have induced a reactive mode in a proactive population.

### The reactive shift in cortical activity

We first identified the specific brain regions associated with cognitive control in the AX-CPT. For this purpose, we performed a meta-analysis of previously reported brain coordinates where the BOLD activity was stronger for B cues than for A cues. The meta-analysis revealed a network of fronto-parietal and occipital areas commonly co-activated during tasks requiring executive functions (Bressler & Menon, 2010; Niendam et al., 2012). The network was more right-lateralized in the middle frontal gyrus. The increased activity to B relative to A cues might indicate one or several processes. First, since the B cue is informative of the response to be chosen (i.e., nontarget), the activity might reflect response preparation. Second, given the high frequency of target trials (approx. 60%–70%), the activity might reflect inhibition or response selection processes recruited to inhibit the target response (Mostofsky & Simmonds, 2008). In the present experiment, we found stronger B cue than A cue activity in frontal, parietal, and occipital areas across load levels, in accordance with the meta-analysis results.

A key finding was that more reactive control was associated with decreased cue-related activity but increased probe-related activity in B trials in prefrontal areas. In particular, right dlPFC showed a shift from cue- to probe-related activity with load in B trials. This finding is consistent with the brain activity patterns found in populations that are typically characterized as reactive, such as older adults and schizophrenia patients, where activity in the right prefrontal cortex is weaker after the cue but stronger after the probe, as compared to healthy young controls (Braver et al., 2009; Edwards et al., 2010; Paxton et al., 2007). The present results are also in agreement with a study using tDCS and the AX-CPT in which cathodal stimulation on the right but not the left dlPFC led to more errors in BX trials (Gómez-Ariza et al., 2017), leading the authors to conclude that the right dlPFC is involved in the mechanism that flexibly adjusts cognitive control. It is likely that several partly independent cognitive control functions are involved in AX-CPT performance, and in the strategic shift in control mode, including encoding, updating, and maintaining goal representations, as well as distractor suppression and the inhibition of inappropriate responses. Each of these functions has been associated with PFC, but the mapping of cognitive functions to specific regions in the PFC is still controversial. For example, although dlPFC has been argued to be involved in goal representations that have modulatory influence on subcortical and posterior cortical regions (D’Ardenne et al., 2012; Miller & Cohen, 2001), others have emphasized the role of dlPFC in response inhibition (Cipolotti et al., 2016; Knoch & Fehr, 2007; Zmigrod, Colzato, & Hommel, 2014). Part of the discrepancy may be due to functional interdependence between PFC regions and their related cognitive functions. For example, suppression of dlPFC function through direct stimulation may impair inhibitory processing indirectly via the inferior PFC (iPFC), which in turn is more directly involved in inhibiting motor programs (Aron, Robbins, & Poldrack, 2014; Eisenegger, Treyer, Fehr, & Knoch, 2008). Also, dlPFC seems to be most strongly associated with inhibition that is conditional on a certain context than with the simple act of stopping (Chikazoe et al., 2009; Jahfari, Stinear, Claffey, Verbruggen, & Aron, 2010). Consistent with this finding, Swann, Tandon, Pieters, and Aron (2013) have shown temporal differences in dlPFC and iPFC activation within a trial: Whereas the dlPFC was activated by task cues, the iPFC was active at the time of action inhibition. According to the DMC framework, the proactive control mode involves the anticipation and prevention of conflict prior to its occurrence, whereas the reactive control mode is characterized by the need to detect and resolve conflict after it has occurred (i.e., after the probe). Thus, the present data may be taken to show that taxing of WM resources leads to reduced reliance on a dlPFC-dependent proactive form of inhibitory function and to increased reliance on stimulus-induced inhibitory functions. However, the present study was designed to reveal a potential load-induced reactive shift in cognitive control but is essentially agnostic regarding the specific information-processing mechanisms (inhibitory or other) that might mediate such a shift.

In summary, our results give support to the notion of dynamic involvement of the dlPFC in the flexible use of cognitive control. This region was recruited at the presentation of the cue when a proactive mode of behavior allowed for preparing a response in advance, or it was engaged after the presentation of the probe when a reactive mode of behavior was used.

### The role of the VTA/SN in context updating

The VTA dopaminergic nuclei have innervations to the prefrontal cortex, and this region’s physiological effects have been related to WM function (Cools & D’Esposito, 2011; D’Esposito & Postle, 2015). Phasic dopamine signals have been proposed to implement a form of reinforcement learning. The phasic release of dopamine acts as a learning signal that is used to predict when rewards will occur (Montague, Dayan, & Sejnowski, 1996). In line with this proposal, it has been reported that dopamine neurons fire in events associated with reward prediction errors—that is, a difference between received and predicted rewards (Schultz, Dayan, & Montague, 1997). According to this view, large reward prediction errors signal an unexpected event. Interestingly, this is exactly the condition under which a gating signal should be released so that the context can be updated in order to adapt to the current opportunities for reward. There is also a learning component to this, as the phasic dopamine release strengthens the likelihood that the signal will occur again under similar circumstances. One possibility is that the gating and learning effects of dopamine could be implemented by the same physiological gain control mechanism (Cohen, Braver, & Brown, 2002; Servan-Schreiber, Printz, & Cohen, 1990). An alternative model proposes that dopamine is used to train the timing of the gating signal, but that the gating signal itself is released from the basal ganglia (Frank et al., 2001). However, both models share the prediction that gating signals and the updating of WM should be accompanied by a phasic release of dopamine.

Hyperactivity in VTA/SN has previously been reported for patients with schizophrenia relative to healthy controls in the AX-CPT (Yoon, Minzenberg, Raouf, D’Esposito, & Carter, 2013), but to the best of our knowledge, a B > A effect has not been reported before. D’Ardenne et al. (2012) showed that VTA/SN was activated only in trials in which representation of the task context was required, but the design of their task did not differentiate between distinctive task-relevant contexts. The present results extend D’Ardenne et al.’s finding by showing that the VTA/SN activity is sensitive to context type—that is, B versus A.

### The role of the LC in context updating

Growing evidence has implicated the LC-NE system in cognition and attention in humans (Robbins & Arnsten, 2009). In general, LC activity is thought to increase neural gain in brain circuits relevant for the task at hand and optimizes “exploitation” of information within the current focus (Aston-Jones & Cohen, 2005; Berridge & Waterhouse, 2003; Eldar, Cohen, & Niv, 2013; Mather, Clewett, Sakaki, & Harley, 2016; Sara, 2009; Usher, Cohen, Servan-Schreiber, Rajkowski, & Aston-Jones, 1999). For example, one recent study showed increased activity in a region matching the location of LC in the incongruent versus congruent trials of the Stroop task (Köhler et al., 2016), and in another study, LC activity was associated with strengthening representations of goal-relevant visual scenes (Clewett, Huang, Velasco, Lee, & Mather, 2018). The LC-NE system influences prefrontal functions through its extensive projections (Aston-Jones & Waterhouse, 2016; Szabadi, 2013). Electrophysiological data obtained from monkeys indicated that LC activity mobilizes the sensory and attentional resources needed for processing of a cue and the energy necessary for subsequent action (Varazzani, San-Galli, Gilardeau, & Bouret, 2015). In short, LC-NE activity seems to mediate cognitive effort through moment-to-moment phasic arousal signals as a function of task engagement.

For the first time, we have reported an involvement of LC in context updating. We found that LC activity was more strongly triggered by B than by A cues, suggesting stronger task engagement for the nontarget conditions. B cue trials (BX + BY: 24%) were less frequent than A cue trials (AX + AY: 76%), and furthermore, the B cues consisted of a set of different silhouette stimuli, whereas the A cue was signaled by one particular silhouette in the low-load condition, or by a set of five silhouettes in the high-load condition. The LC is known to be sensitive to stimulus saliency—for instance, as defined by novelty (Aston-Jones & Cohen, 2005; Corbetta, Patel, & Shulman, 2008; Sara & Bouret, 2012). Thus, the relative “novelty” of B cues could potentially yield a more potent driving signal to LC neurons. The lack of a significant cue type effect in the high-load condition in the LC could be taken to support this interpretation, since the relative “novelty” of B cues should be less in this condition. However, behavioral data from AX-CPT experiments in which stimulus novelty has been controlled (Chatham et al., 2009) or trial type frequency has been manipulated (Redick, 2014; Richmond et al., 2015) do not indicate that the novelty or base frequency of trial types in itself plays a major role in the relative weighting of proactive versus reactive processing modes.

The processing of B cues allows for proactive preparation of the nontarget response, since the predictive validity of B cues is 100%. Thus, the B > A effect observed in the LC might be related to generation of the effort needed for proactive response preparation. Finally, stronger brain activity to B cues has previously been associated with inhibition of a target response that might already be prepared on the basis of the high frequency of target trials (MacDonald & Carter, 2003). However, the exact source of the stronger B-cue-related activity could involve elements of some or all of the processes suggested above, and it remains to be resolved by future studies. Taken together, the results suggest that the LC may be recruited in situations that demand cognitive control in order to facilitate response preparation.

### Individual differences and modulation of cortical and brain stem activity

The exploratory analysis of individual differences revealed that proactive participants (i.e., those who had positive PBI scores in the low-load condition) had significant B > A cue effects in the right dlPFC, and larger B-cue-related activity in LC in low than in high load. The lower B-related activity paralleled the trend toward a load-induced reduction of the PBI score in this group, suggesting that proactive participants flexibly engaged a more reactive processing mode under high load. This arguably constituted a more efficient strategy than actively sustaining the high-load information. This is because sustaining the high-load cue information would be more computationally costly than sustaining low-load cue information. The predictive values of the cue information were similar across load conditions (i.e., equal trial-type frequencies), and the utility of sustaining the high-load cue information should therefore have been lower. According to this processing economics perspective, a reactive shift appears to be an adaptive adjustment. Consistent with this idea, proactive individuals had similar performance levels (total accuracy) across load conditions and had a significant reactive shift in right dlPFC and LC activity. At the other extreme, reactive participants showed overall larger cue-related activity in the right dlPFC and LC, and no differences with load. Overall enhanced brain activity has been found in older adults during the delay period of the AX-CPT (Paxton et al., 2007). One possibility is that the reactive group did not adapt their strategy to changes in the task conditions and indiscriminately invested more resources in the task. Furthermore, a trend toward a reduction in accuracy (e.g., worse performance in BX and also in AY trials) resembles the pattern observed in a study with monkeys (Blackman, MacDonald, & Chafee, 2013). In that study, monkeys, who overall showed reactive behavior in the standard AX-CPT, showed even more reactive behavior (i.e., reduced BX accuracy) when they were administered ketamine, but also a reduction of accuracy in AY trials. Although comparison between species should be done with caution, the striking similarity of this pattern with the one observed in our reactive participants suggests that the context deficits caused by load involve neuromodulatory mechanisms of glutamatergic synapses (Mather et al., 2016). Overall reactive behavior does not seem to imply that proactive strategies were not applied on a trial-by-trial basis. For instance, the reactive group showed similar RT slowing in AY trials; however, a reactive mode prevailed. This is consistent with a recent theory suggesting that LC is at the core of the interaction between higher-order areas and the variability that exists between individuals (Unsworth & Robison, 2017). The results from our intermediate group are more difficult to interpret, and it can be argued that they, similarly to the reactive group, did not significantly change their strategy with increasing load.

In a recent behavioral study by Wiemers and Redick (2018), young healthy individuals with relatively low WMC employed a more reactive processing mode than did high-WMC participants. However, in a split-half analysis of the trials, the high-WMC group exhibited a relatively stable processing strategy, and the low-WMC group became more proactive over the course of the experiment, as their experience with it developed. Moreover, an analysis of AX RT distributions revealed significant RT differences between WMC groups only for the 10% slowest trials, suggesting that the reactive processing mode is characterized by a limited number of attentional lapses, consistent with the ideas presented in Unsworth and Robison (2017). One interesting possibility is that the occurrence of attentional lapses might be related to the amount of available WM resources per se, and could be seen not only in individuals with low WMC, but in any sample following increased WM load. Furthermore, if reactive processing is due to occasional attentional lapses, then a proactive shift should be associated with a reduced tendency for attentional lapses, especially in BX trials. Consistent with this notion, increased WM load (but not perceptual load) is associated with increased distractibility (Lavie, 2005), and both proactive processing and a reduced tendency toward attentional lapses can be induced by specific training (Braver et al., 2009; deBettencourt, Cohen, Lee, Norman, & Turk-Browne, 2015; Gonthier et al., 2016). However, it is currently not clear that there is a relation between a shift in control mode and a tendency toward attentional lapses. Wiemers and Redick (2018) evaluated the proactive shift as a function of increases in AY error rate and decreases in BX error rate, but due to the need for a relatively large number of trials for them to perform the analysis, attentional lapses were defined by long RTs on AX trials. Furthermore, whether the frequency of attentional lapses was reduced as a function of proactive shift in low-WMC participants was not reported. In the present study, the load manipulation affected BX accuracy, but it did not have any measurable impact on RTs. It is thus unclear whether occasional attentional lapses are associated with limitations in WM resources in general, or more specifically with WMC, and whether proactive or reactive control shifts are regulated by the occurrence of attentional lapses.

### Limitations

Some limitations should be acknowledged. First, study of the small nuclei of the brain is problematic, due to low signal-to-noise ratios (Brooks, Faull, Pattinson, & Jenkinson, 2013) and high variability in brain stem structures between individuals. To overcome this limitation, we employed a neuromelanin-sensitive structural scan, which allowed us to locate with relatively high accuracy the position of the LC (Keren et al., 2009; Langley, Huddleston, Liu, & Hu, 2017; Tona et al., 2017). Second, the silhouettes used here constitute a modification of the commonly used letters version of the AX-CPT; however, this proved to be a valid modification of the AX-CPT and gave results similar to those reported previously with other versions [employing letters, dots (Jones et al., 2010), line drawings (Lucenet & Blaye, 2014), words (Paxton et al., 2007), and cartoons (Chatham et al., 2009)]. Altogether, the data suggest that the cognitive control mechanisms involved in this task are largely independent of the type of stimuli, although they are probably dependent on specific visual circuits. Third, the use of event-related designs has some drawbacks, such as correlation between regressors (Mumford, Poline, & Poldrack, 2015) and lower sensitivity. Here, we used jittering with an average interstimulus interval of 3 s. This interval is reasonable, and the jittering has been deemed a good strategy to reduce collinearity between adjacent trials, which was convenient here for comparing the different trial types. Fourth, conclusions on intersubject variability in neurophysiological activity are often limited due to reduced statistical power or task limitations (Hedge, Powell, & Sumner, 2018). One study on human adults investigated interindividual variations in a motivational factor (i.e., reward rate) using the AX-CPT and showed that this factor correlated with activation in the fronto-polar cortex (Locke & Braver, 2008). Our results suggest that different physiological processes may underlie interindividual variation in strategies, and therefore the assessment of variability within groups of healthy adults, especially if they are used as a control group, may allow us to better disentangle independent processes.

## Conclusions

The present study has shown that WM context load plays an important role in the variability of cognitive control mode within individuals. Furthermore, the cortical BOLD data support the idea that the right dlPFC is a hub in context updating; activity in this region is activated by cues in the proactive mode, and by probes in the reactive mode. Additionally, the brain stem BOLD data support the idea that both the dopaminergic and noradrenergic systems are involved in updating context representations. Although the data do not allow for strong conclusions about the specificity of each system, the results seem generally consistent with the notion that the dopaminergic system mediates a gating signal to the control network, whereas the noradrenergic system may be involved in dynamic regulation of the level of task engagement. Finally, the results may indicate that one source of the observed reactive mode of control in developmental and neuropsychiatric groups can be traced back to limitations in WM capacity, potentially due to dysregulation of WM input gating and/or cognitive effort.

## Electronic supplementary material

ESM 1 (PDF 1.05 mb)
