# Supplementary material for: Task context load induces reactive cognitive control: An fMRI study on cortical and brain stem activity
Source: Cogn Affect Behav Neurosci. 2019 Jan 18;19(4):945–65. doi: 10.3758/s13415-019-00691-6 (PMC6711881; doi:10.3758/s13415-019-00691-6)
Supplement: Supplementary file 1 — (PDF 1.05 mb) [file 13415_2019_691_MOESM1_ESM.pdf]

Supplementary material

1. Appendix A: web-based experiments, behavioral analysis on young adults only

130 participants were included (mean age: 31.2 (SD: 7.1), age range: 19-47; 39 female). The resulting samples for each load level were: low, N = 45; medium, N = 41; and high, N = 44.

Average accuracy was higher in low load as compared to intermediate and high load (average accuracy low load: 0.96 (0.03), intermediate load: 0.92 (0.08), high load: 0.92 (0.07); U test low vs intermediate: Z = 2.45, p = 0.014; low vs high: Z = 2.86, p = 0.004). There was a main effect of condition on accuracy in all levels of load (Friedman test, main effect of condition: p <0.001), with more errors in AY trials than in AX and BY trials (Wilcoxon test, p <0.001) and more errors in BX than in AX and BY trials (Wilcoxon test, p <0.001). Under high load, participants committed more errors in BX than AY trials (Wilcoxon test, Z = 2.23, p = 0.025) but not under low or intermediate load (p = 0.595 and p = 0.658, resp.). When comparing between load levels, accuracy was lower under high load compared to low load for BX and BY trials (U test, Z = 2.30, p = 0.021, and Z = 2.69, p = 0.007, resp.). Regarding RTs, participants were overall slower in the high load condition (t test low vs high: t = 2.21, p = 0.033; intermediate vs high: t = 2.09, p = 0.041). AY trials were significantly slower than all other trials (trial type effect: F(3,387) = 32.68, p <0.001) (see Figure 3). The context-d' was significantly lower in high load compared to low load (context-d' high load: 3.39, low load: 4.02; U test, Z = 2.58, p = 0.010).

The context-d' of intermediate load was not significantly different from the other load conditions (context-d' intermediate load: 3.73; U test, p >0.2).

2. Appendix B: web-based experiments, behavioral measures

The proactive bias index was significantly lower in high load as compared to low load (Kruskal-Wallis test for main load effect: H = 6.18, p = 0.045; post-hoc independent samples tests: load 1 vs 3: Z = 2.54, p = 0.011; load 1 vs 2: Z = 0.69, p = 0.48; load 2 vs 3: Z = 1.41, p = 0.158). There was a significantly higher frequency of reactive participants in load 3 as compared to load 1 (Pearson chi-squared: 11.97, p = 0.003). These results indicate that the load manipulation successfully induced a reduction in the use of proactive control.

To further characterize the strategies at the different load levels, we calculated the correlation between RTs in non-target trials (Table S1, Chatham et al. 2009). We found that RT partial correlation between AY and BY trials (controlling for RT in AX trials) was significant only in high load (rho = 0.37, p = 0.007). We note that the difference between the correlation coefficients at the different load levels was not significant (low vs. high load: Z = 1.35, p = 0.08). On the other hand, RTs partial correlation between BX and BY trials was significant in all load levels (low load: partial rho = 0.66, p <0.001; intermediate load: partial rho = 0.80, p <0.001; high load: partial rho = 0.59, p <0.001). This points towards a more reactive regime under high load, where probe-driven processing is related to high correlation between responses to Y probes. Although the opposite was expected to happen in B trials, we found a significant correlation between RTs in BX and BY trials

in all load levels. A possible reason may be a dissociation between proactive and reactive modes of control, where probe-driven control was recruited with load, without affecting cue-driven control.

Table S1: Partial correlation between RTs in non-target trials (AY-BY and BX-BY) controlling for RT in AX trials.

Web-based experiments			
	Low load	Intermediate load	High load
AY-BY	0.098	0.244	0.373*
BX-BY	0.662*	0.8*	0.592*

fMRI experiments			
	Low load		High load
AY-BY	-0.12		0.489*
BX-BY	0.18		0.147

*: significant correlation.

3. Appendix C: fMRI experiment, behavioral measures

To further explore the strategies at the different load levels, we calculated the RT partial correlation between non-target trials (controlling for RT in AX trials). The partial correlation between RTs of AY and BY trials was significant only in high load (rho = 0.489, p = 0.006, Table S1). This correlation coefficient was significantly higher than the correlation coefficient in low load (rho low load: -0.121; Z = 2.32, p = 0.01). On the other hand, RT partial correlation between BX and BY trials was not significant in any load level, and not significantly different between each other. This points towards

a more reactive regime under high load, where response times to the Y probe were significantly correlated.

The context sensitivity measure d' decreased with load although the results were marginally significant ($F = 3.21$, $p = 0.084$, e.s. 0.10; Table S2).

We identified subgroups of participants with proactive, reactive or intermediate behavioral trends. In Table S2 we display the accuracy, context-d' and proactive index, for the proactive, reactive and intermediate subgroups at low and high load. The proactive bias index was significantly lower in high load as compared to low load in proactive and intermediate participants (interaction load by group: $F(2,28) = 9.36$, $p = 0.001$; post-hoc t tests load 1 vs 3: proactive group:$t(8) = 2.84$, $p = 0.022$; intermediate: $t(12) = 3.17$, $p = 0.008$).

The groups significantly differed in the context-d' ($F = 15.39$, $p < 0.001$, e.s. = 0.52), with reactive group having significantly smaller context-d' than intermediate and proactive ($p < 0.001$; Table S2). Table S3 shows the accuracy per trial type and group.

Table S2: Mean and standard deviation (SD) of accuracy, context-d' and proactive-bias index for the identified groups at low and high load.

Mean accuracy (SD)		
Group	Low load	High load
Proactive	0.96 (0.01)	0.96 (0.03)
Intermediate	0.98 (0.02)	0.97 (0.04)
Reactive	0.92 (0.07)	0.88 (0.09)
Mean	0.96 (0.04)	0.94 (0.06)

context-d' (SD)		
Group	Low load	High load
Proactive	4.26 (0.35)	3.95 (0.57)
Intermediate	4.11 (0.67)	3.67 (0.91)
Reactive	2.75 (0.82)	2.57 (1.03)
Mean	3.76 (0.91)	3.43 (1.01)

Proactive Index		
Group	Low load	High load
Proactive	0.92	0.42
Intermediate	0	-0.43
Reactive	-0.87	-0.34
Mean	0.01	-0.15

Table S3: Accuracy per trial for the identified groups at low and high load.

Accuracy per trial								
	Low load				High load			
Group	AX	AY	BX	BY	AX	AY	BX	BY
Pro.	0.98	0.86	0.99	1	0.98	0.88	0.96	0.99
Int.	0.98	0.98	0.98	0.98	0.98	0.98	0.91	1
Reac.	0.96	0.97	0.78	0.98	0.96	0.85	0.72	0.97

Table S4: Coordinates from previous AX-CPT studies incorporated in the meta-analysis.

**Study**	**N subjects**	**Coordinate (MNI)**
Perlstein et al. 2003	15	47 46 14
		-29 50 21
		-40 30 21
		-46 7 20
Lesh et al. 2013	54	-50 -70 50
		52 12 36
		50 -44 50
		-48 32 24
		-44 -52 42
		34 -68 44
		56 28 28
		2 22 48
Edwards et al. 2010	14	-58 3 11

Continued on next page

Table S4 – continued from previous page

Study	N subjects	Coordinates (MNI)
		-53 7 26
		50 15 26
		41 4 22
		57 7 29
		28 3 55
		-22 4 53
D'Ardenne et al., 2012	19	39 52 28
Poppe et al. 2016	56	46 -66 -14
		42 -68 -16
		-38 -80 -12
		-34 -82 -12
		28 -74 36
		-46 -48 50
		-30 -42 -24
		-40 -60 -14
		32 16 54
		24 20 54
		-28 -84 18
		-42 38 22
		28 54 18
		-40 -4 12
		-32 14 50
		52 -44 48
Poppe et al. 2015	22	-52 20 24

Continued on next page

Table S4 – continued from previous page

Study	N subjects	Coordinates (MNI)
		50 22 24
		-1 55 27
		-2 6 24
		-2 -65 21
		-32 -61 52
		39 24 44
		49 22 30
		49 -45 48
		4 28 47
Lopez Garcia et al. 2015	26	48 12 26
		32 22 0
		-16 -2 64
		44 -50 -18
		30 -68 44
		-40 -54 -14
		-26 -70 40
		-46 4 26
		40 -80 -8
		28 -64 38
		-34 -84 -8
		-22 -72 54
		52 14 30
		-56 8 32
Paxton et al. 2008	16	27 35 47

Continued on next page

Table S4 – continued from previous page

Study	N subjects	Coordinates (MNI)
		46 22 24
		47 35 32
		-36 51 26
		40 22 -14
		56 23 -7
		53 43 -9
		-24 16 13
		-59 16 8
		-51 29 -14
		43 11 30
		58 11 33
		33 -3 60
		-1 5 67
		-48 -1 42
		18 -7 56
		-29 -6 54
Yoon et al. 2008	24	-3 -72 12
		-45 52 12
		45 17 32
		21 -72 48
		-3 34 40

Table S5: Coordinates in resulting AX-CPT meta-analysis

Cluster	Hemisp.	Area	Coordinate (MNI)
1	RH	IFG/MFG/SMA	48 16 30
2	RH	IFG	34 22 4
3	LH	MFG/SMA	-24 0 56
4	LH	IFG/MFG	-48 6 24
5	LH	IPL	-44 -50 46
6	RH	IPL	50 -44 48
7	LH	FG	-40 -58 -14
8	RH	IOL	42 -72 -14
9	LH	IOL	-34 -82 -10
10	LH	Precuneus	-2 -68 16
11	RH	Precuneus	30 -70 42

RH: rigth hemisphere; LH: left hemisphere; SMA: supplementary motor area; IFG: Inferior Frontal Gyrus; MFG: Middle Frontal Gyrus; FG: fusiform gyrus; IPL: inferior parietal lobe; IOL: inferior occipital lobe.

Table S6: Main and interaction effects: cue type and load.

	Region	BA	Size	Coords	Z
CUE TYPE EFFECT					
RH	MFG	46	15	50 30 18	2.83
	ACC		14	4 22 42	2.68
LH	FG	37	271	38 -50 -20	4.12
LH	Occipital		143	-42 -66 -16	3.75
LH	IPL	40	19	-42 -48 56	3.22
LOAD EFFECT					
RH	SMG	40	42	56 -36 42	3.44
LH	PA	6	8	-56 2 28	2.48*
CUE BY LOAD EFFECT					
RH	MFG	9	27	52 20 32	2.79
RH	PA	6	10	52 4 44	2.45*
LH	FG		4	-40 -76 -14	2.69

RH: rigth hemisphere; LH: left hemisphere; ACC: anterior cingulate cortex; MFG: Middle Frontal Gyrus; PA: precentral area; SMG: supramarginal gyrus; FG: fusiform gyrus; IPL: inferior parietal lobe. Clusters surviving small volume correction on regions obtained from the meta-analysis, $p < 0.005$, unc. * small volume correction, $p < 0.01$, unc. Cluster size in voxels.

Table S7: Main and interaction effects: probe type and load

	Region	BA	Size	Coords	Z
PROBE TYPE EFFECT					
RH	PA	6	508	48 8 30	5.83
LH	PA	6	479	-52 2 38	4.58
RH	IFG	47	160	36 22 -10	4.31
LH	MFG	9	68	-38 34 32	4.20*
RH	ACC		10	2 14 46	3.42*
RH	IPL	40	288	44 -46 44	4.62
LH	IPL	40	467	-30 -58 46	4.38
RH	FG	37	683	44 -52 -16	6.61
LH	Cerebellum		222	-40 -64 -18	5.45
LOAD EFFECT					
No significant effects					
PROBE BY LOAD EFFECT					
RH	MFG	9	84	48 22 32	3.19*
RH	PA	6	17	52 4 44	3.03*
LH	PA	6	5	-54 -4 30	2.63*
LH	Cerebellum		5	-32 -62 -14	2.97*

RH: rigth hemisphere; LH: left hemisphere; ACC: anterior cingulate gyrus; MFG: Middle Frontal Gyrus; PA: precentral area; IPL: inferior parietal lobe; FG: Fusiform Gyrus. Clusters surviving small volume correction on regions obtained from the meta-analysis, $p < 0.001$, unc. * small volume correction, $p < 0.005$, unc. Cluster size in voxels.

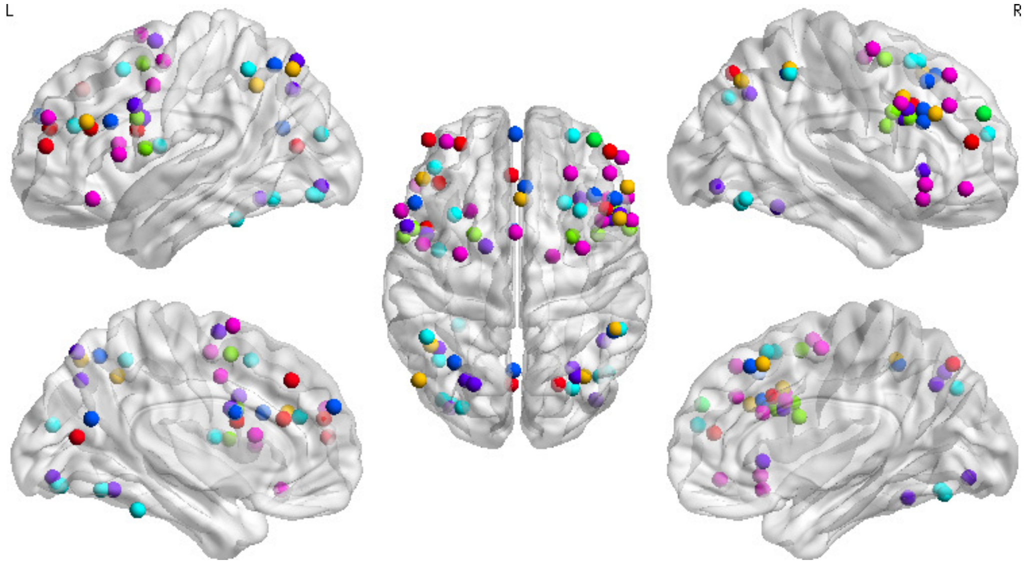

Figure S1: Coordinates extracted from fMRI studies on AX-CPT, B >A contrast.

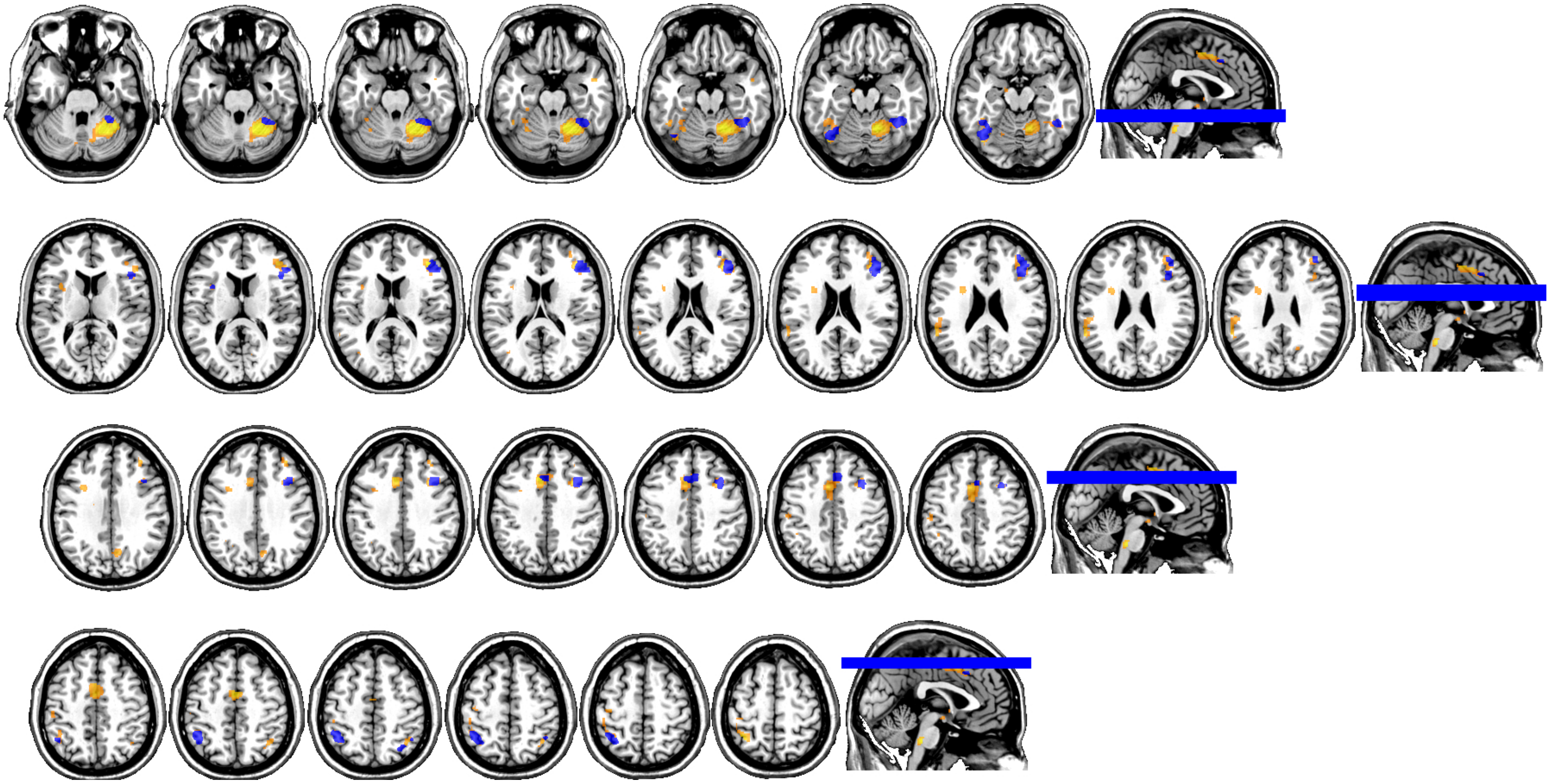

Figure S2: B >A contrast as in Figure 4, bottom. The regions in blue fall within the meta-analysis mask used for small volume correction.
